# Supplementary material for: Cyclodextrin-based host-guest complexes loaded with regorafenib for colorectal cancer treatment
Source: Nat Commun. 2021 Feb 3;12:759. doi: 10.1038/s41467-021-21071-0 (PMC7858623; doi:10.1038/s41467-021-21071-0)
Supplement: Supplementary file 1 — Supplementary Information [file 41467_2021_21071_MOESM1_ESM.pdf]

Cyclodextrin-based host-guest complexes loaded with regorafenib for colorectal cancer treatment

Supplementary Information

Hongzhen Bai[1], Jianwei Wang[1], Chi Uyen Phan[1], Qi Chen[1], Xiurong Hu[1], Guoqiang Shao[2], Jun Zhou[1], Lihua Lai[3]*, Guping Tang[1]*

Corresponding to: Lihua Lai, lailihua@zju.edu.cn
Guping Tang, tangguping@zju.edu.cn

[1]Department of Chemistry, Zhejiang University, 310028 Hangzhou, PR China
[2]Department of Nuclear Medicine, Nanjing First Hospital, Nanjing Medical University, 210029 Nanjing, PR China
[3]Department of Pharmacology, School of Medicine, Zhejiang University, 310058 Hangzhou, PR China

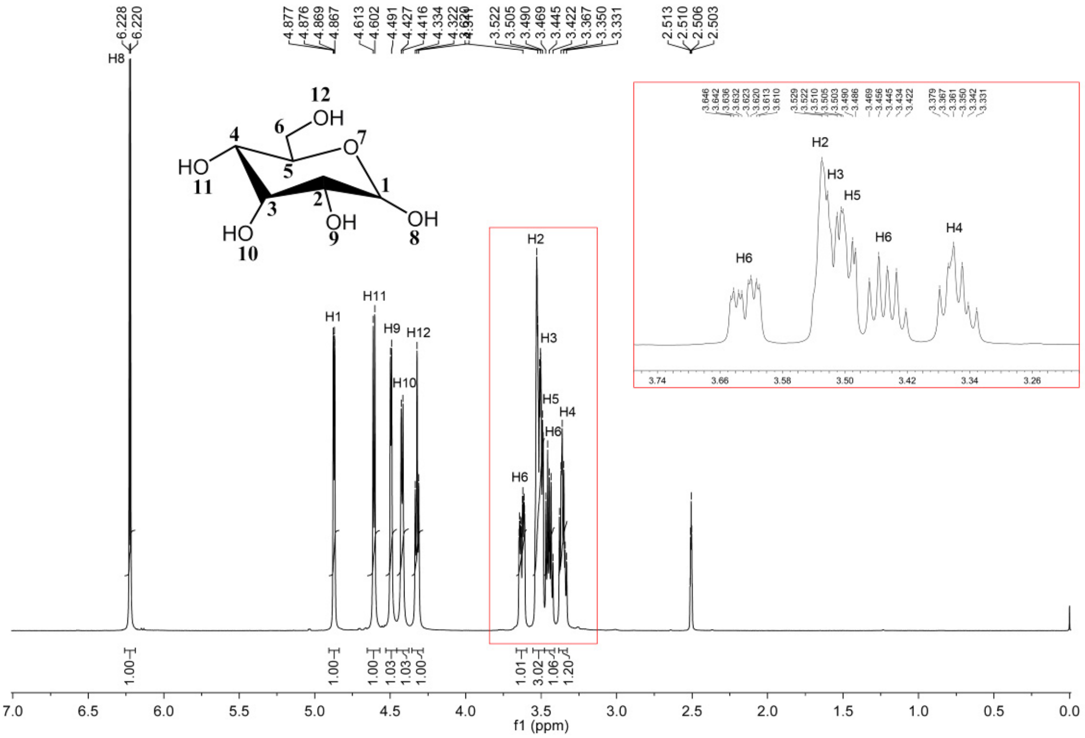

Supplementary Figure 1. ^{1}H NMR spectrum of Mannose (500 MHz, DMSO-D6, room temperature). The NMR assay was conducted for the used compounds using DMSO-D6 as solvent.

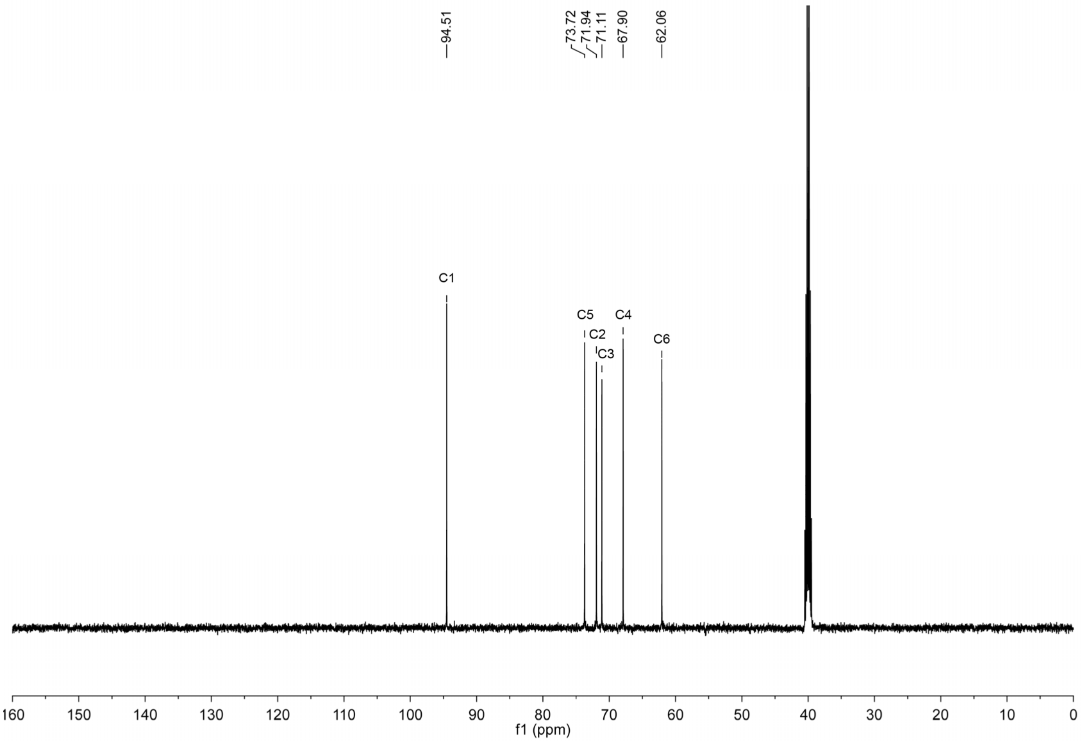

Supplementary Figure 2. ^{13}C NMR spectrum of Mannose (500MHz, DMSO-D6, room temperature).

^{1}H NMR (500 MHz, DMSO-D6) δ 6.224 (d, J = 4.4 Hz, 1H), 4.872 (dd, J = 4.3 Hz,

0.8 Hz, 1H), 4.607 (d, *J* = 5.5 Hz, 1H), 4.495 (d, *J* = 4.1 Hz, 1H), 4.421 (d, *J* = 5.7 Hz, 1H), 4.316 (d, *J* = 5.7 Hz, 1H), 3.628 (ddd, *J* = 11.1 Hz, 5.1 Hz, 1.7 Hz, 1H), 3.526 (d, *J* = 3.6 Hz, 1H), 3.517 (dd, *J* = 9.3 Hz, 3.1 Hz, 1H), 3.510-3.486 (m, 1H), 3.456-3.422 (m, 1H), 3.355 (td, *J* = 9.1 Hz, 5.5 Hz, 1H). ^{13}C NMR (126 MHz, DMSO-D6) δ 94.51, 73.72, 71.94, 71.12, 67.90, 62.06.

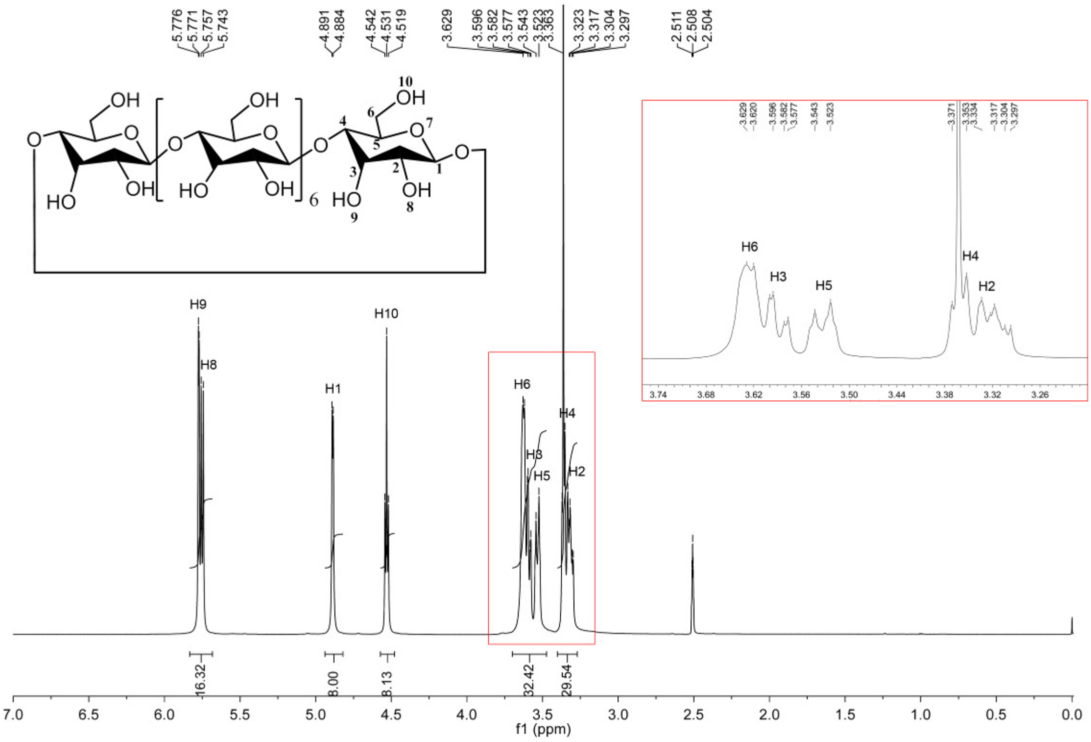

Supplementary Figure 3. ^{1}H NMR spectrum of γ-CD (500 MHz, DMSO-D6, room temperature).

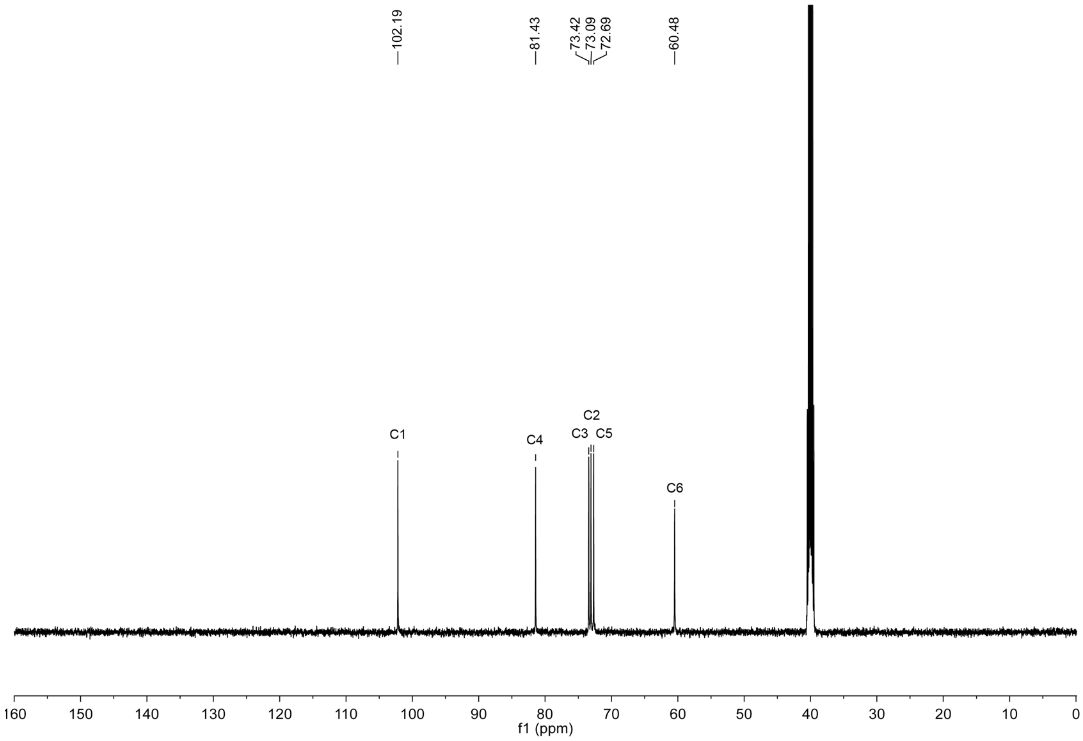

Supplementary Figure 4. ^{13}C NMR spectrum of γ-CD (500 MHz, DMSO-D6, room temperature).

^{1}H NMR (500 MHz, DMSO-D6) δ 5.773 (d, J = 2.5 Hz, 1H), 5.750 (d, J = 7.0 Hz,

1H), 4.888 (d, *J* = 3.5 Hz, 1H), 4.531 (t, *J* = 5.6 Hz, 1H), 3.625 (d, *J* = 4.6 Hz, 2H), 3.589 (dd, *J* = 9.3 Hz, 2.2 Hz, 1H), 3.533 (d, *J* = 9.8 Hz, 1H), 3.371-3.353 (m, 1H), 3.334-3.297 (m, 1H). ^{13}C NMR (126 MHz, DMSO-D6) δ 102.19, 81.43, 73.42, 73.09, 72.69, 60.48.

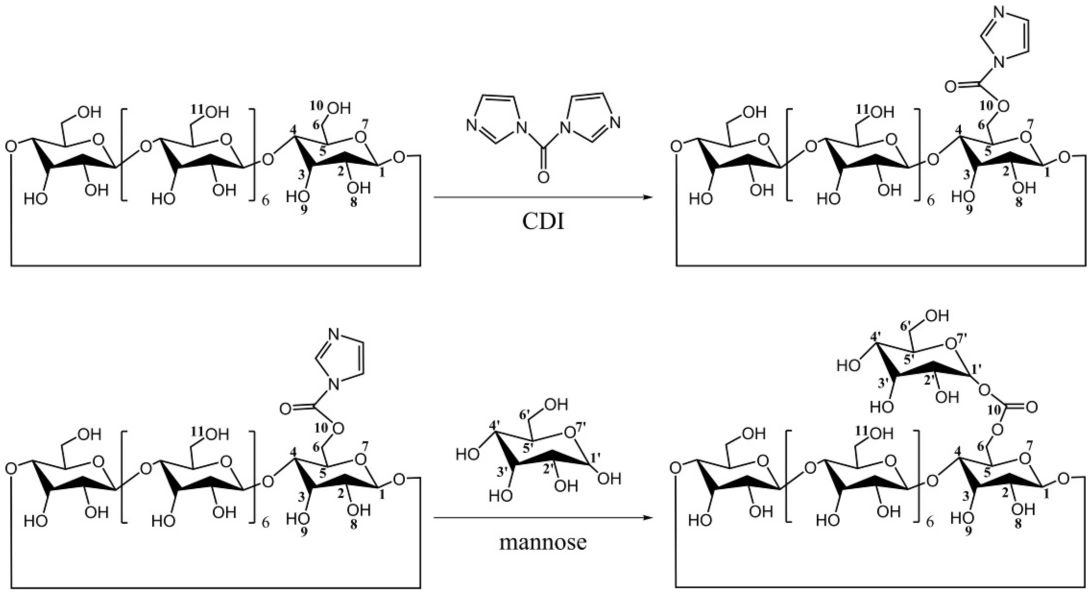

Supplementary Figure 5. Schematic diagram of synthetic route of M-γ-CD.

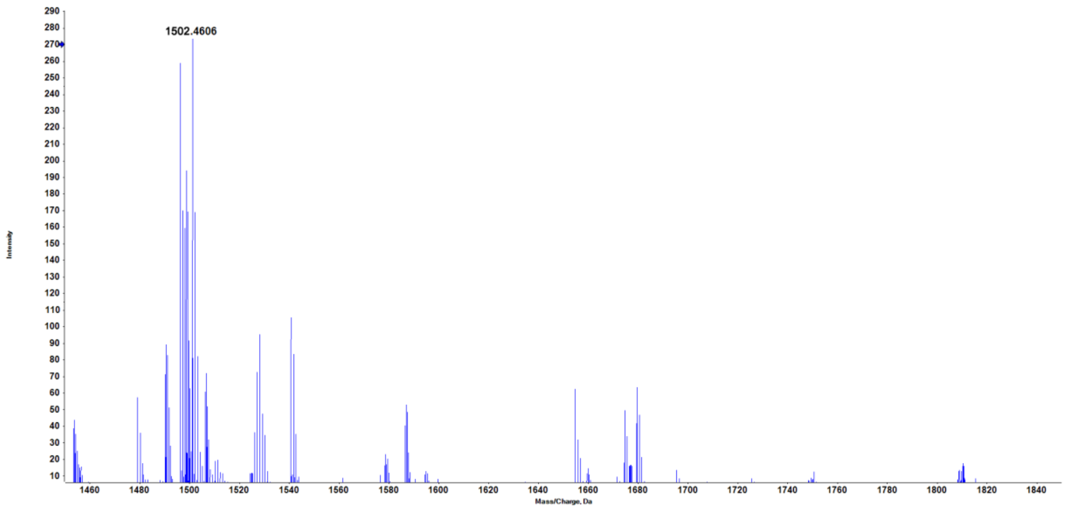

Supplementary Figure 6. ESI-TOF-MS spectrum of M-γ-CD.

ESI-ToF-MS: calculated for M-γ-CD requires *m/z* 1502.4623. M-γ-CD, $C_{55}H_{90}O_{47}$: $12.0000 \times 55 + 1.0078 \times 90 + 15.9949 \times 47 = 1502.4623$, found *m/z* 1502.4606.

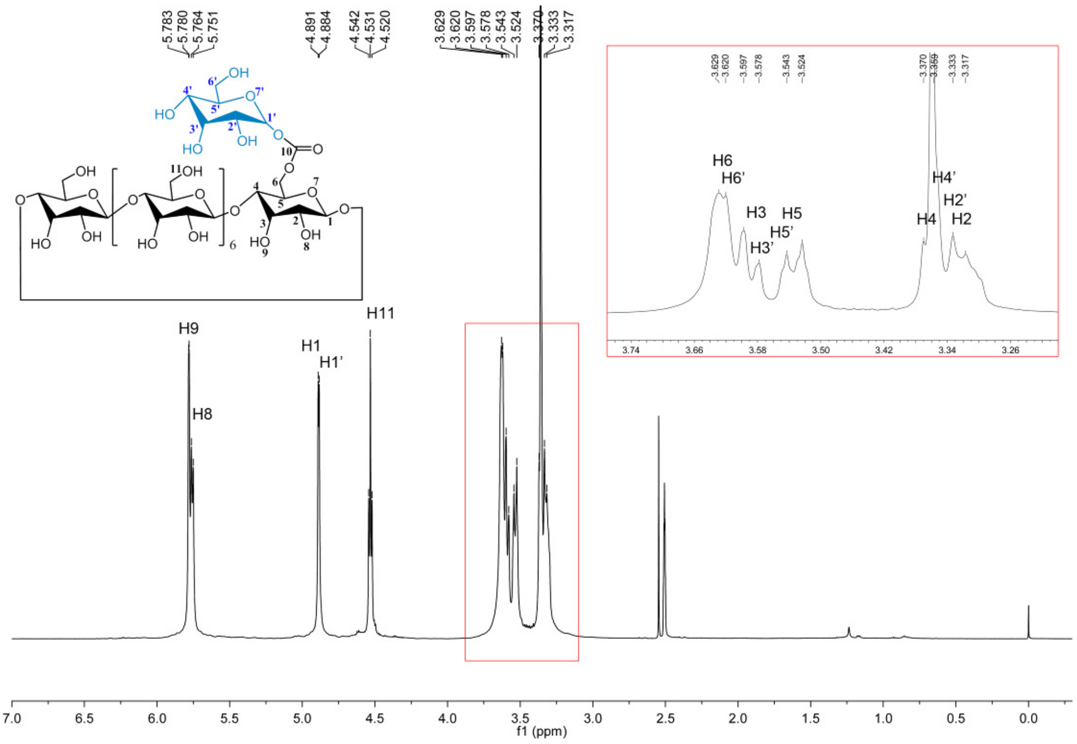

Supplementary Figure 7. ^{1}H NMR spectrum of M-γ-CD (500 MHz, DMSO-D6, room temperature).

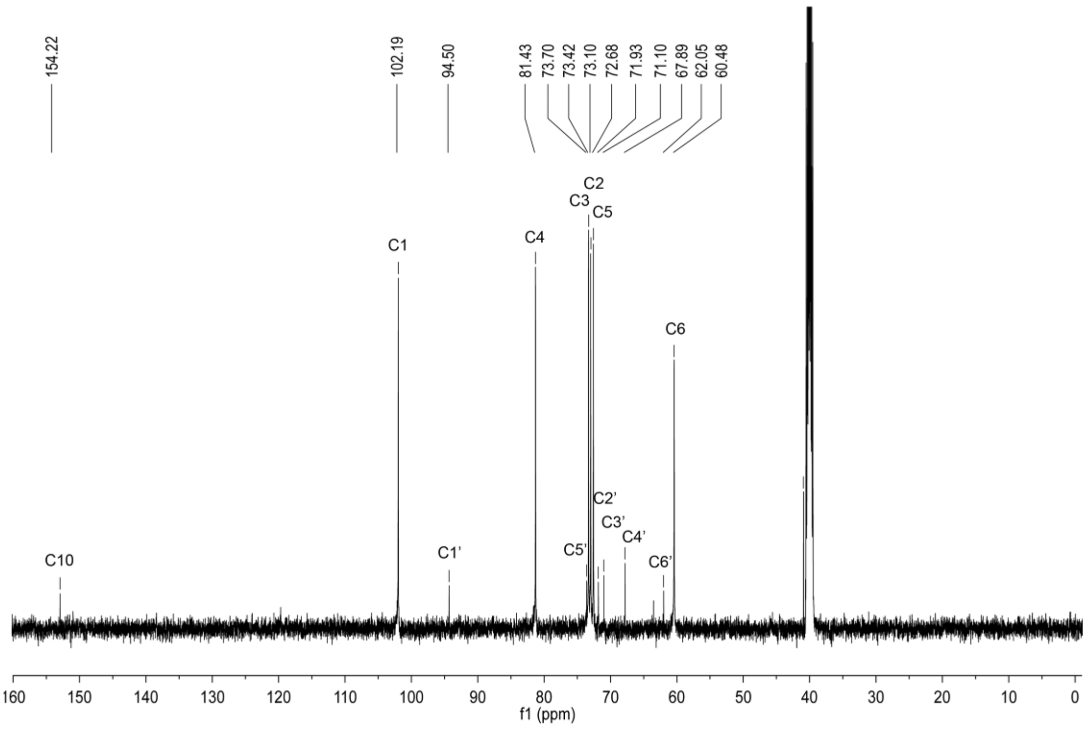

Supplementary Figure 8. ^{13}C NMR spectrum of M-γ-CD (500 MHz, DMSO-D6, room temperature).

^{1}H NMR (500 MHz, DMSO) δ 5.781 (d, *J* = 1.7 Hz, 1H), 5.758 (d, *J* = 6.4 Hz, 1H),

4.887 (d, J = 3.5 Hz, 1H), 4.884 (s, 1H), 4.531 (t, J = 5.6 Hz, 1H), 3.625 (d, J = 4.3 Hz, 2H), 3.620 (s, 2H), 3.588 (d, J = 9.6 Hz, 1H), 3.578 (s, 1H), 3.543 (s, 1H), 3.533 (d, J = 9.8 Hz, 1H), 3.370-3.333 (m, 1H), 3.370-3.359 (m, 1H), 3.333 (s, 1H), 3.325 (d, J = 8.1 Hz, 1H). ^{13}C NMR (126 MHz, DMSO) δ 154.22, 102.19, 94.50, 81.43, 73.70, 73.42, 73.10, 72.68, 71.93, 71.10, 67.89, 62.05, 60.48.

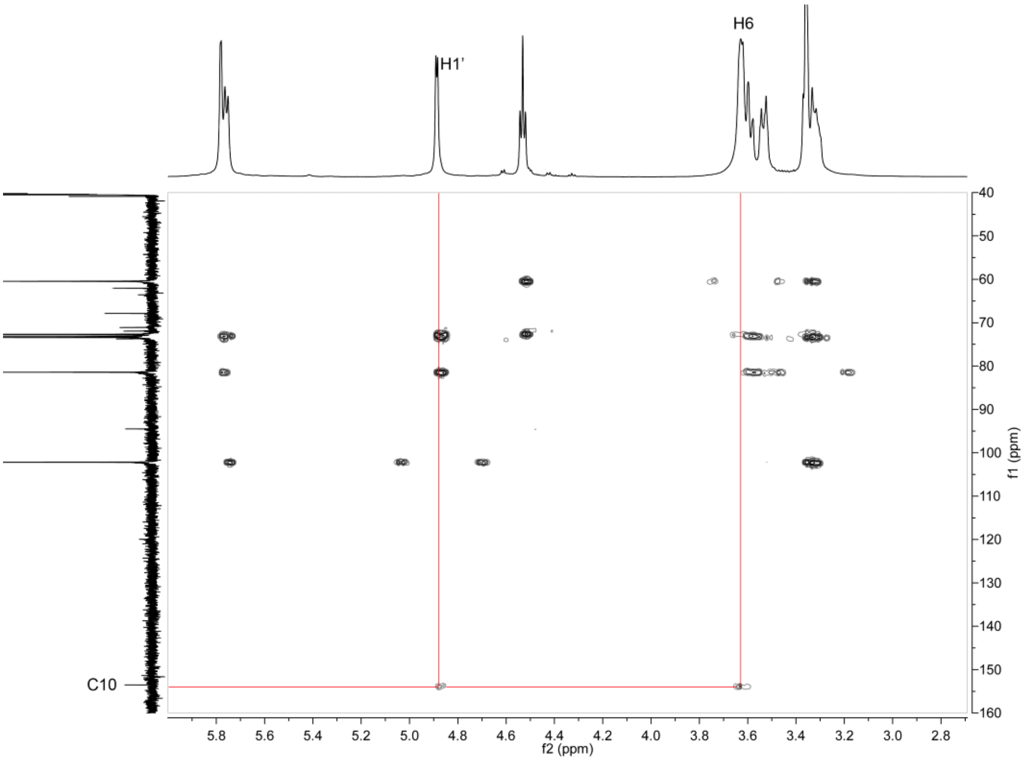

Supplementary Figure 9. HMBC spectrum of M-γ-CD (500 MHz, DMSO-D6, room temperature).

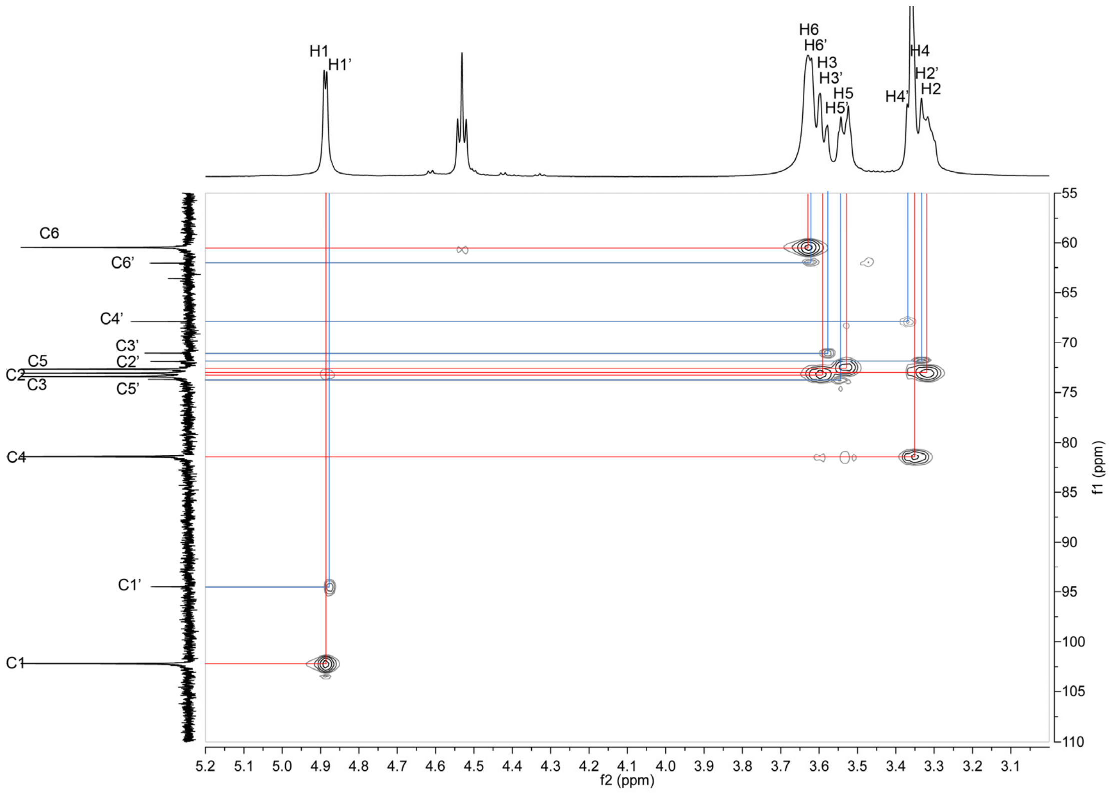

Supplementary Figure 10. HSQC spectrum of M-γ-CD (500 MHz, DMSO-D6, room temperature).

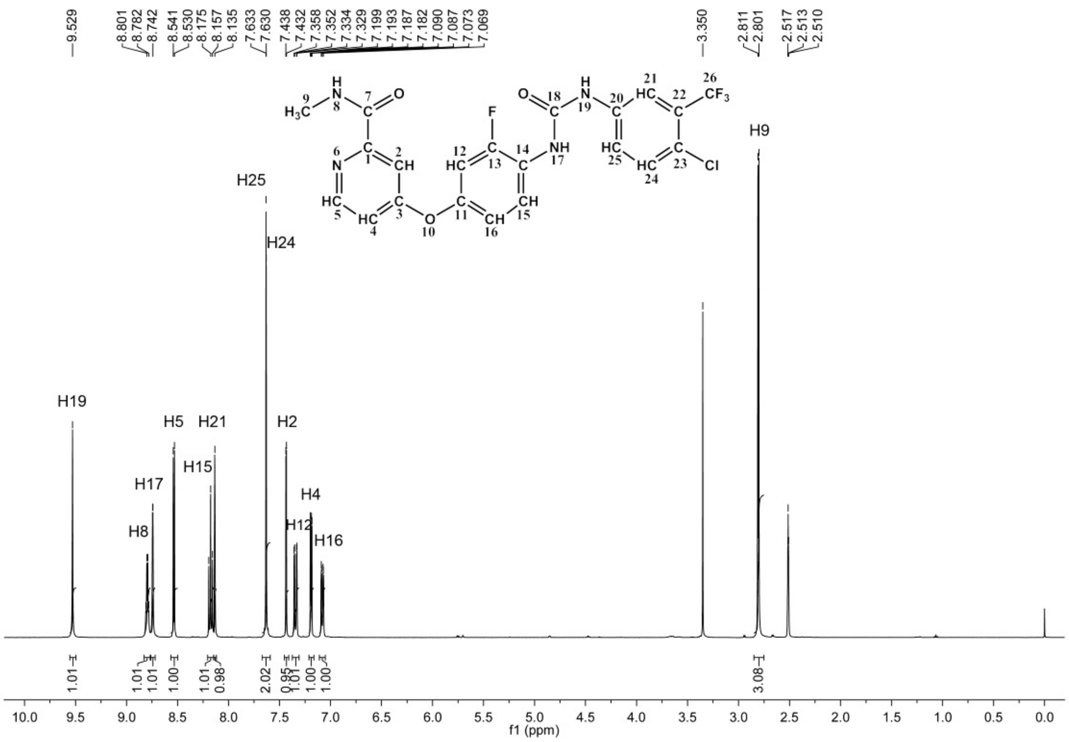

Supplementary Figure 11. ^{1}H NMR spectrum of RG (500 MHz, DMSO-D6, room temperature).

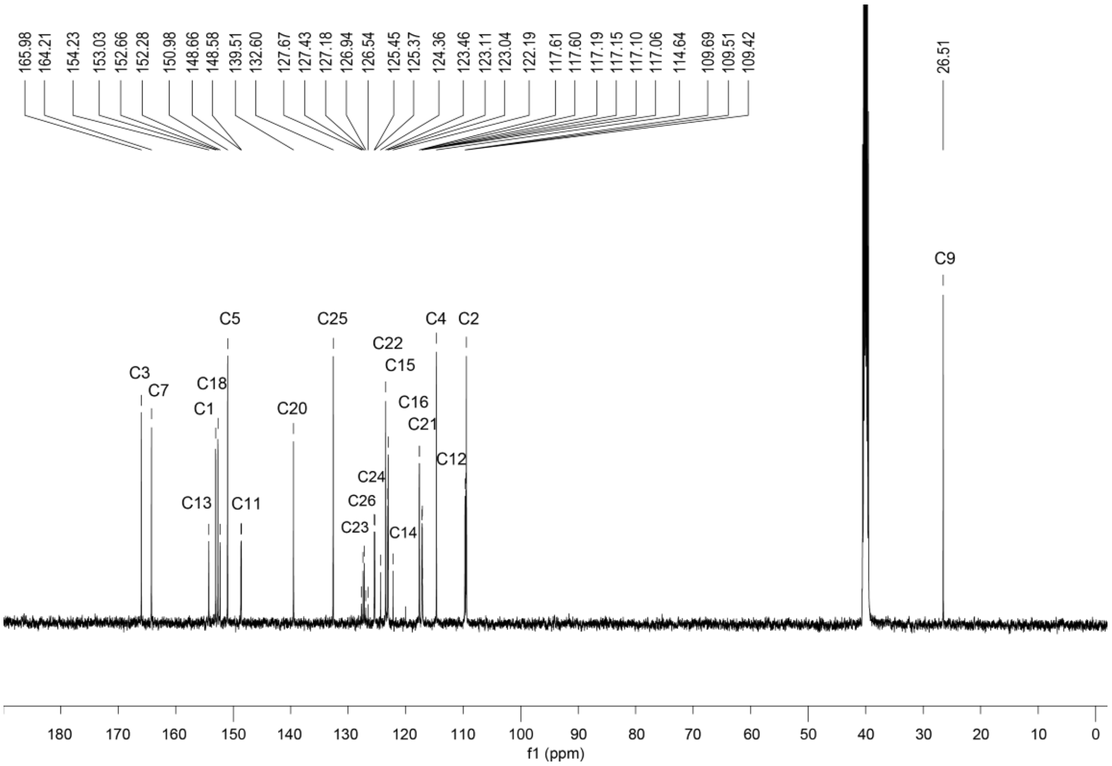

Supplementary Figure 12. ^{13}C NMR spectrum of RG (500 MHz, DMSO-D6, room temperature).

^{1}H NMR (500 MHz, DMSO) δ 9.529 (s, 1H), 8.796 (q, J = 4.6 Hz, 1H), 8.743 (d, J = 1.6 Hz, 1H), 8.536 (d, J = 5.6 Hz, 1H), 8.175 (t, J = 9.1 Hz, 1H), 8.135 (s, 1H), 7.633

(s, 1H), 7.630 (s, 1H), 7.435 (d, J = 2.6 Hz, 1H), 7.343 (dd, J = 11.6, 2.7 Hz, 1H), 7.190 (dd, J = 5.6 Hz, 2.6 Hz, 1H), 7.080 (dd, J = 8.9 Hz, 1.7 Hz, 1H), 2.806 (d, J = 4.9 Hz, 3H). ^{13}C NMR (126 MHz, DMSO) δ 165.98, 164.21, 154.23, 153.03, 152.66-152.28, 150.98, 148.66-148.58, 139.51, 132.60, 127.66-126.93, 126.54, 125.45-125.37, 124.36-123.46, 123.11, 123.04-122.19, 117.61-117.60, 117.19-117.06, 114.64, 109.69, 109.51-109.42, 26.51.

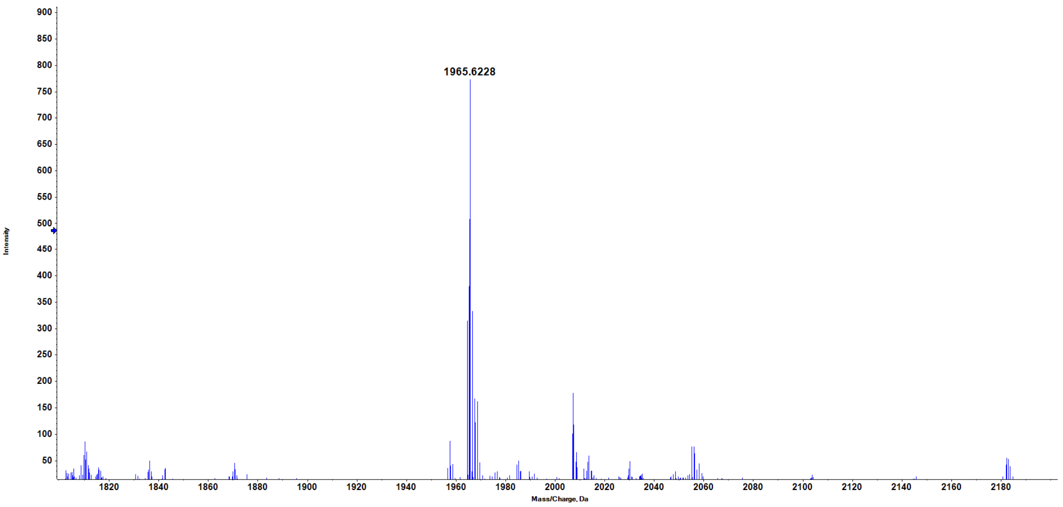

Supplementary Figure 13. ESI-TOF-MS spectrum of RG@M-γ-CD. Since inclusion complexes were non-covalent molecular systems, syringe pump with flow rate of 20 L/min was applied in MS analysis to avoid damage to inclusion complexes.

ESI-ToF-MS: calculated for RG@M-γ-CD requires *m/z* 1984.5384. RG@M-γ-CD, $C_{76}H_{105}ClF_4N_4O_{50}$ [M]: 12.0000 × 76 + 1.0078 × 105 + 34.9688 + 18.9984 × 4 + 14.0030 × 4 + 15.9949 × 50 = 1984.5384. Calculated for $[M-H_2O-H]^-$ requires *m/z* 1965.5201, found *m/z* 1965.6228.

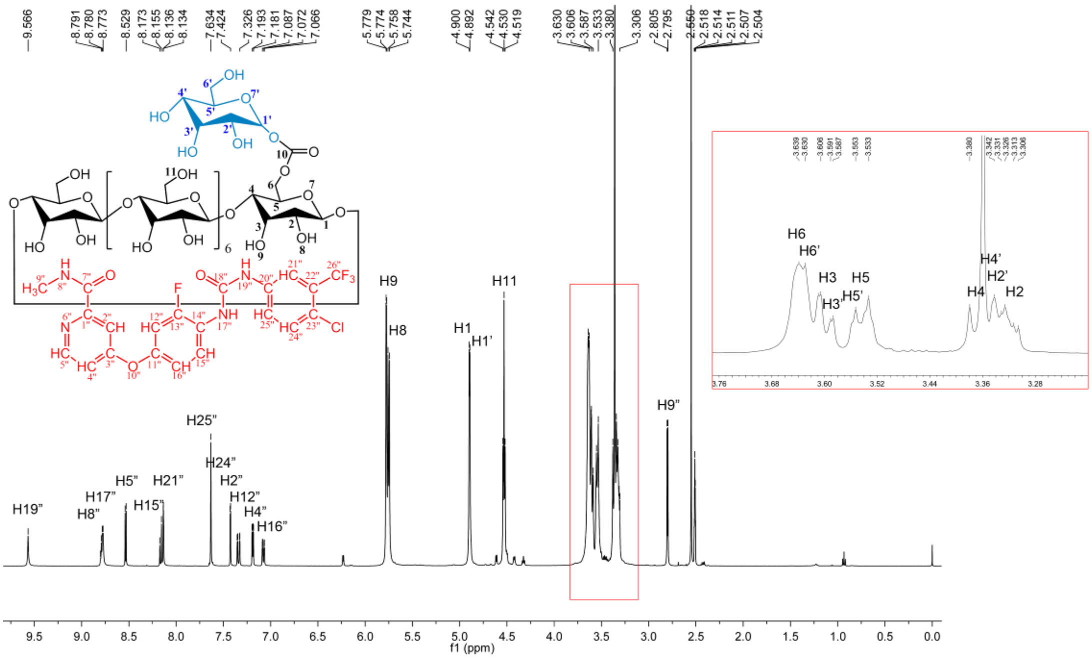

Supplementary Figure 14. ^{1}H NMR spectrum of RG@M-γ-CD (500 MHz, DMSO-D6, room temperature).

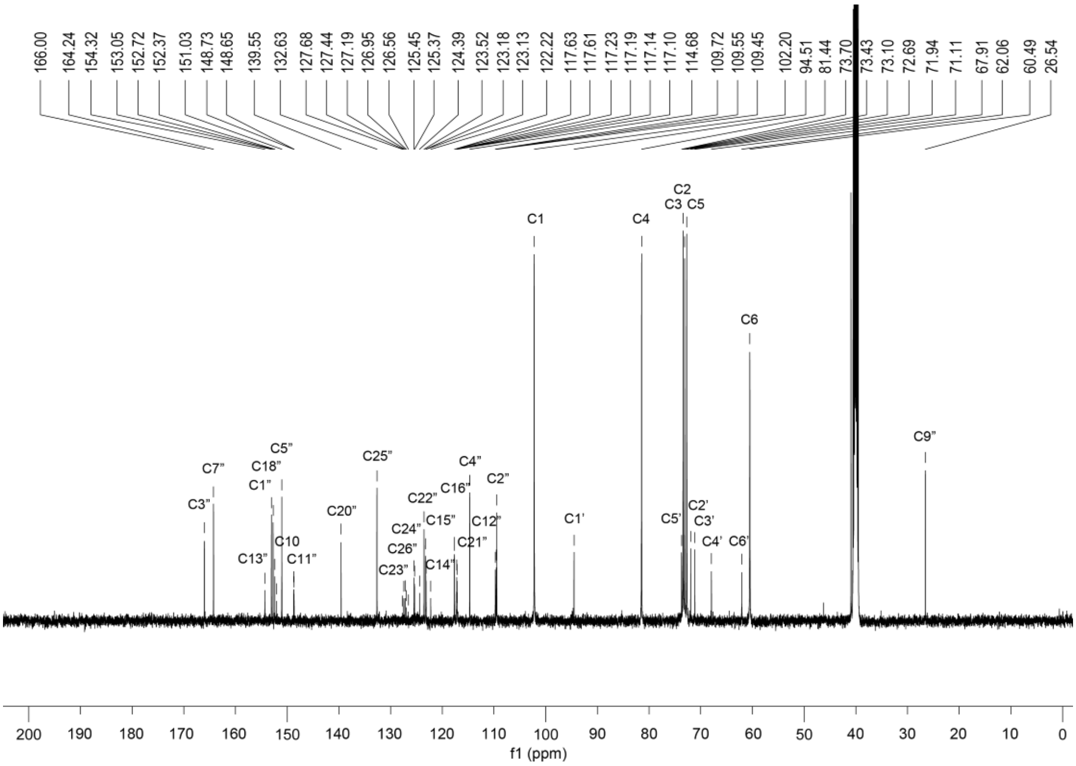

Supplementary Figure 15. ^{13}C NMR spectrum of RG@M-γ-CD (500 MHz, DMSO-D6, room temperature).

^{1}H NMR (500 MHz, DMSO-D6) δ 9.566 (s), 8.795 (d, *J* = 4.6 Hz), 8.777 (d, *J* = 3.6 Hz), 8.534 (d, *J* = 5.6 Hz), 8.164 (d, *J* = 9.0 Hz), 8.135 (d, *J* = 1.1 Hz), 7.634 (s), 7.426 (d, *J* = 2.6 Hz), 7.340 (dd, *J* = 11.6 Hz, 2.7 Hz), 7.190 (dd, *J* = 5.6 Hz, 2.6 Hz), 7.089-7.066 (m), 5.776 (d, *J* = 2.3 Hz), 5.751 (d, *J* = 7.0 Hz), 4.896 (d, *J* = 3.6 Hz), 4.892 (s), 4.530 (t, *J* = 5.6 Hz), 3.634 (d, *J* = 4.6 Hz), 3.630 (s), 3.598 (dd, *J* = 9.4 Hz, 1.8 Hz), 3.589 (d, *J* = 1.9 Hz), 3.553 (s), 3.543 (d, *J* = 9.8 Hz), 3.533 (s), 3.369 (d, *J* = 10.3 Hz), 3.380-3.342 (m), 3.342-3.306 (m), 3.319 (dd, *J* = 9.6 Hz, 3.2Hz), 2.800 (d, *J* = 4.8 Hz). ^{13}C NMR (126 MHz, DMSO-D6) δ 166.00, 164.24, 154.32, 153.05, 152.72, 152.37, 151.03, 148.73-148.65, 139.55, 132.63, 127.68-126.95, 126.56, 125.45-125.37, 123.52, 123.18-123.13, 122.22, 117.63-117.61, 117.19-117.14, 114.68, 109.72-109.55, 109.45, 102.20, 94.51, 81.44, 73.70, 73.43, 73.10, 72.69, 71.94, 71.11, 67.91, 62.06, 60.49, 26.54.

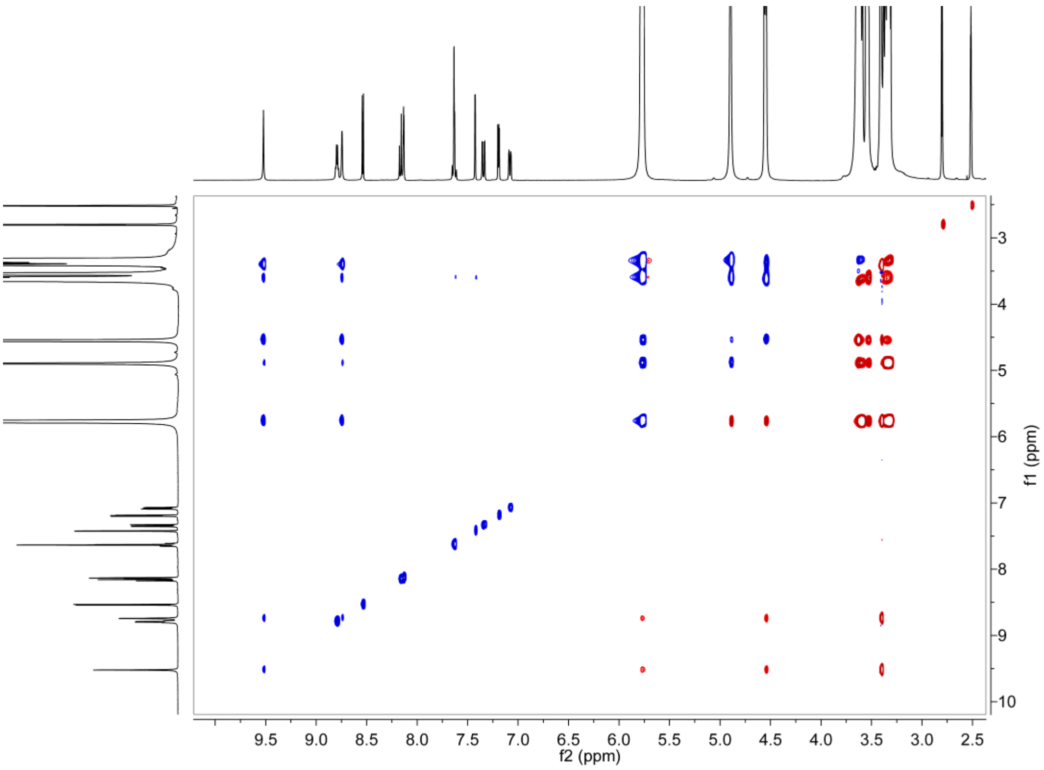

Supplementary Figure 16. 2D-NOESY spectrum (500 MHz, DMSO-D6, room temperature) of RG@M-γ-CD. Supplementary Figure 16 refers to Figure 1d.

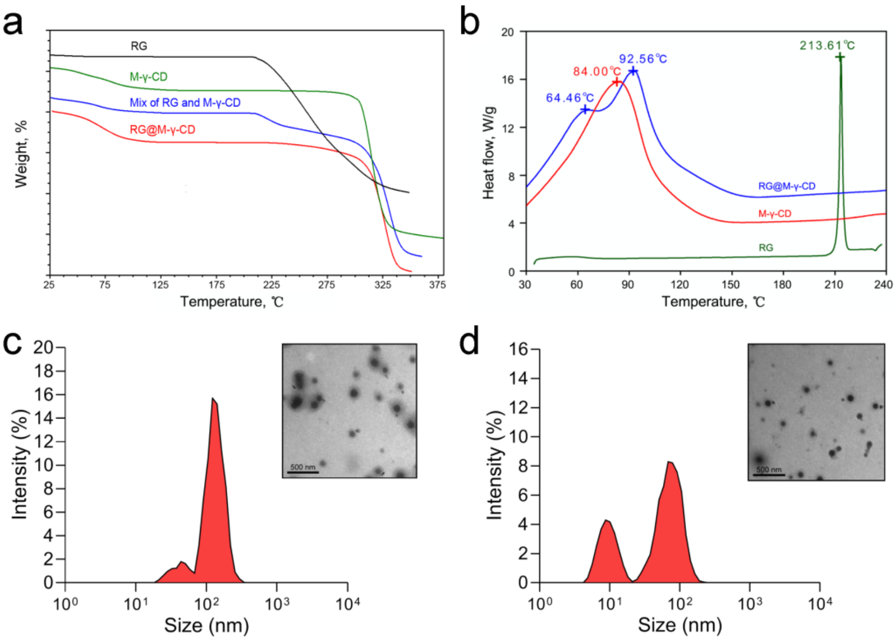

Supplementary Figure 17. After assembly into CNPs, RG@M-γ-CD nano-system was characterized with XRD and TG-DSC. The stability of RG@M-γ-CD CNPs was assessed by DLS, TEM in multiple conditions (PBS with 10% serum or simulated digestive juice). (**a**, **b**) TG and DSC analysis of RG@M-γ-CD. (**c**) DLS measurement and TEM observation of RG@M-γ-CD CNPs after 24 h of incubation in serum-contained PBS. (**d**) DLS measurement and TEM observation of RG@M-γ-CD CNPs after 24 h of incubation in simulated digestive juice.

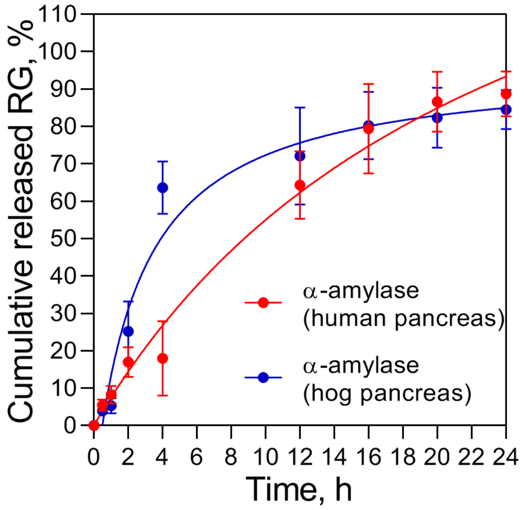

Supplementary Figure 18. Cumulative drug release profile of RG@M-γ-CD CNPs in the presence of α-amylase (100 U/mL). N = 3 biological replicates in each group. Data were expressed as means ± SD. Supplementary Figure 18 refers to Figure 1k.

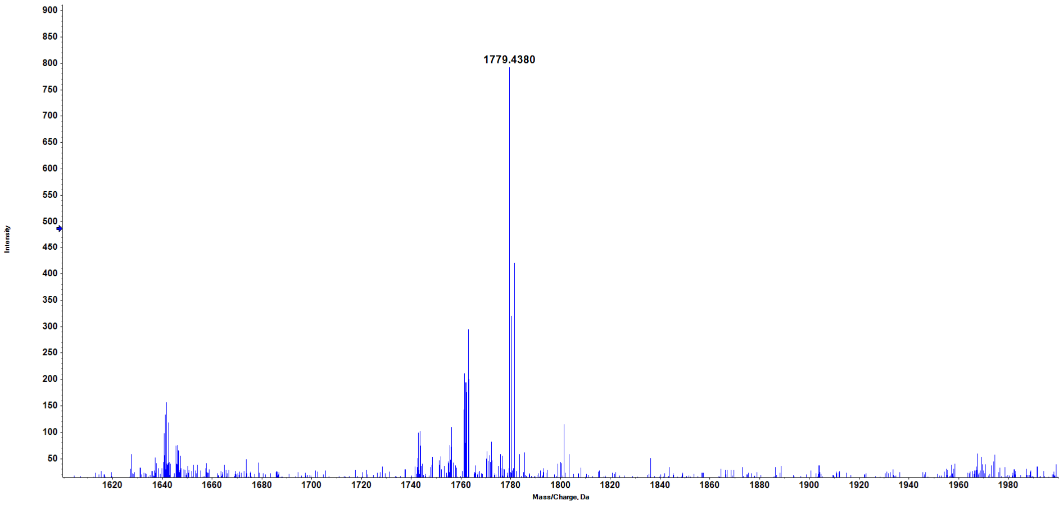

Supplementary Figure 19. ESI-TOF-MS spectrum of RG@γ-CD. In this study, other inclusion complexes and their CNPs were also prepared as controls, including RG@γ-CD, Rhodamine@γ-CD (Rho@γ-CD) and Rho@M-γ-CD. Since inclusion complexes were non-covalent molecular systems, syringe pump with flow rate of 20 L/min was applied in MS analysis to avoid damage to inclusion complexes.

ESI-ToF-MS: calculated for RG@γ-CD requires *m/z* 1778.4943. RG@γ-CD, $C_{69}H_{95}ClF_4N_4O_{43}$ [M]: 12.0000 × 69 + 1.0078 × 95 + 34.9688 + 18.9984 × 4 + 14.0031×4 + 15.9949×43=1778.4947. Calculated for $[M+H]^+$ requires *m/z* 1779.5025, found *m/z* 1779.4380.

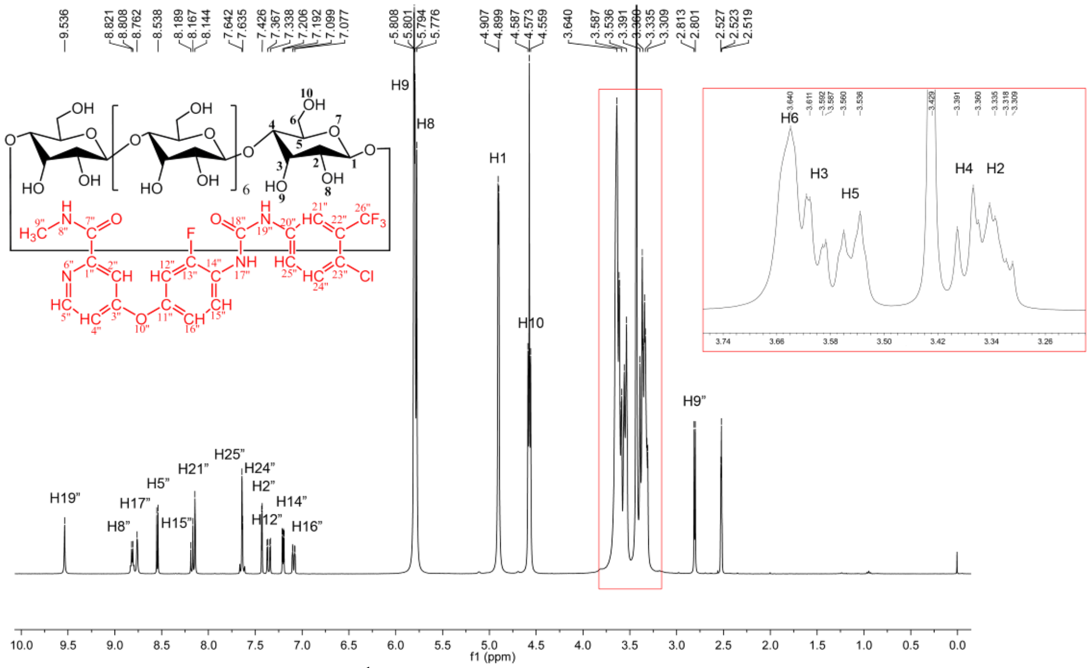

Supplementary Figure 20. ^{1}H NMR spectrum of RG@γ-CD (500 MHz, DMSO-D6, room temperature).

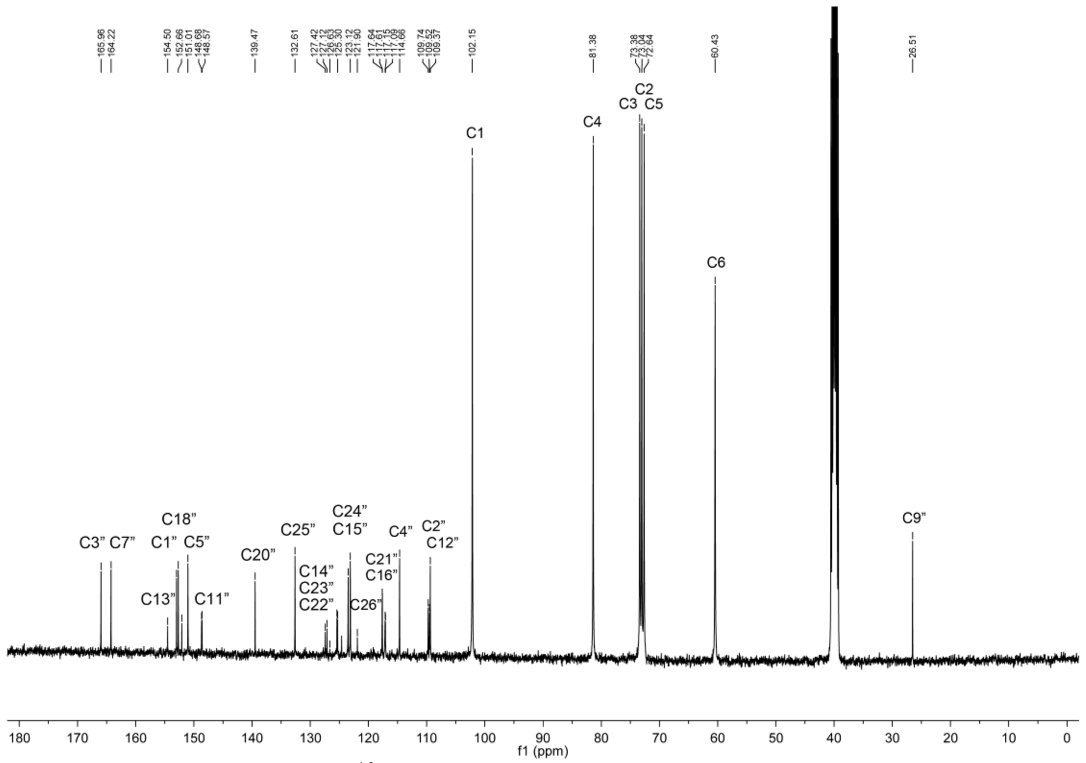

Supplementary Figure 21. ^{13}C NMR spectrum of RG@γ-CD (500 MHz, DMSO-D6, room temperature).

^{1}H NMR (500 MHz, DMSO) δ 9.536 (s), 8.814 (q, *J* = 4.6 Hz), 8.762 (s), 8.545 (d, *J* = 5.6 Hz), 8.178 (d, *J* = 9.1 Hz), 8.144 (s), 7.635 (s), 7.642 (s), 7.429 (d, *J* = 2.6 Hz), 7.356 (dd, *J* = 11.6 Hz, 2.6 Hz), 7.202 (dd, *J* = 5.6 Hz, 2.6 Hz), 7.090 (dd, *J* = 8.9 Hz, 1.5 Hz), 5.805 (d, *J* = 2.4 Hz), 5.785 (d, *J* = 7.1 Hz), 4.903 (d, *J* = 3.4 Hz), 4.573 (t, *J* = 5.6 Hz), 3.640 (s), 3.611-3.587 (m), 3.548 (d, *J* = 9.8 Hz), 3.391-3.360 (m), 3.326 (dd, *J* = 10.1 Hz, 3.6 Hz), 2.807 (d, *J* = 4.9 Hz). ^{13}C NMR (126 MHz, DMSO) δ 165.96, 164.22, 154.50, 152.97, 152.66-152.06, 151.01, 148.68-148.57, 139.47, 132.61, 127.42-127.12, 126.63, 125.41-125.30, 123.49, 123.12, 121.90, 117.64-117.61, 117.15-117.09, 114.66, 109.74-109.37, 109.52, 102.15, 81.38, 73.38, 73.04, 72.64, 60.43, 26.51.

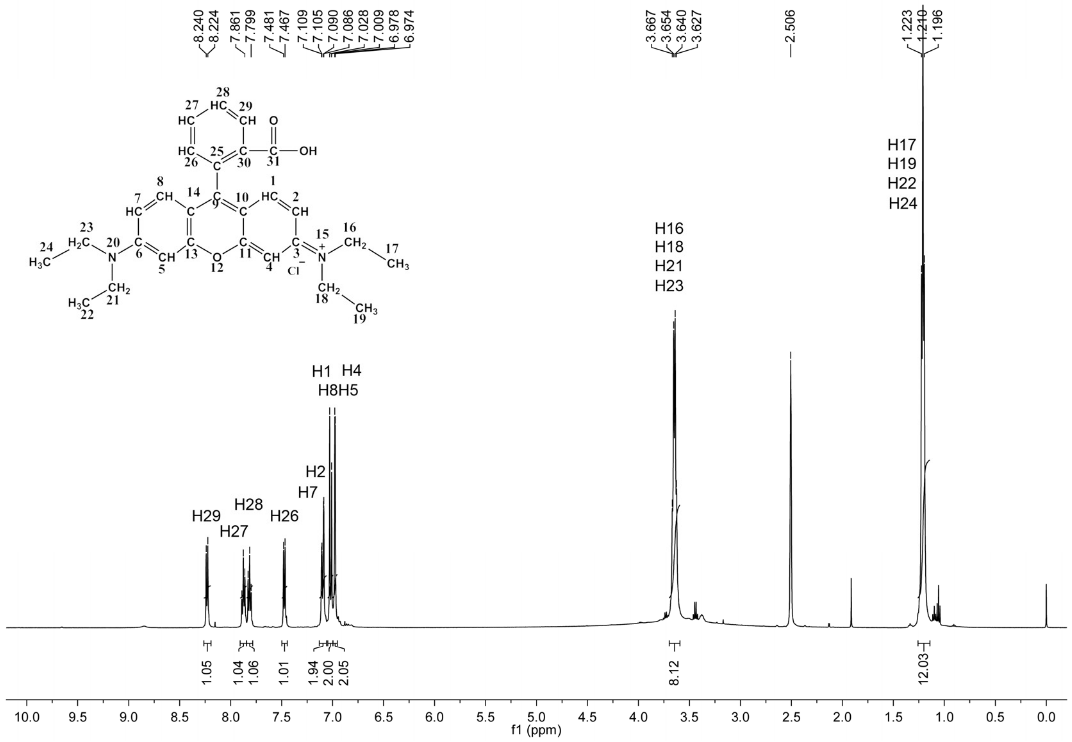

Supplementary Figure 22. ^{1}H NMR spectrum of Rho (500 MHz, DMSO-D6, room temperature).

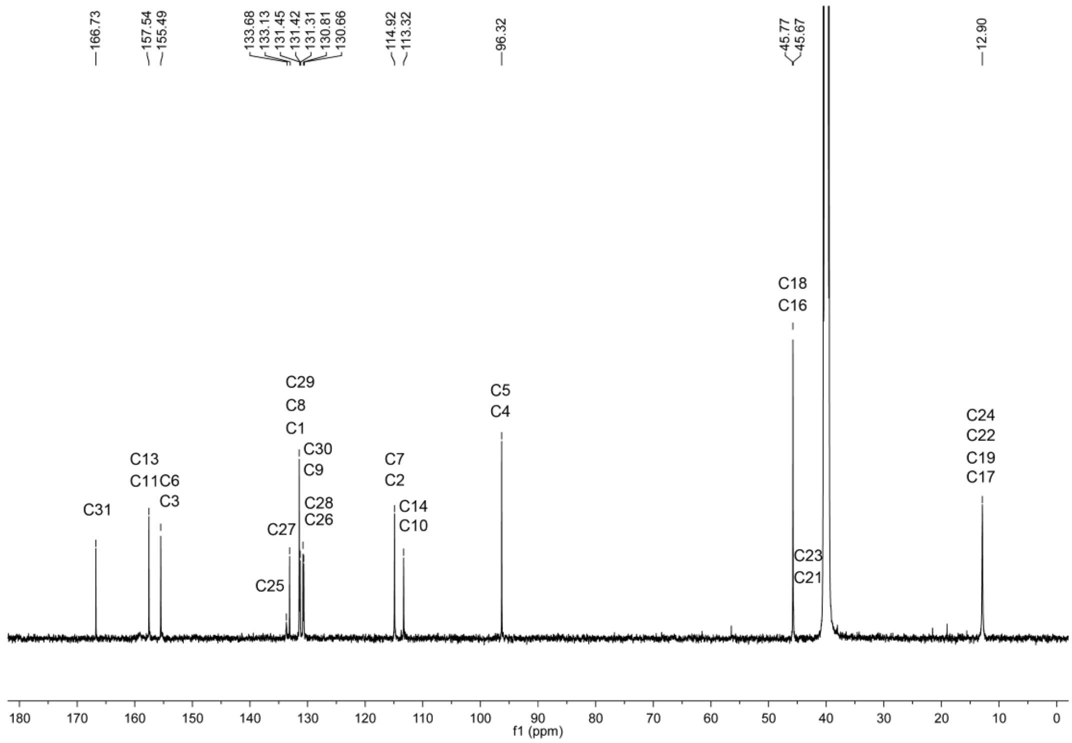

Supplementary Figure 23. ^{13}C NMR spectrum of Rho (500 MHz, DMSO-D6, room temperature).

^{1}H NMR (500 MHz, DMSO) δ 8.232 (d, J = 7.6 Hz, 1H), 7.875 (t, J = 7.2 Hz, 1H),

7.814 (t, *J* = 7.4 Hz, 1H), 7.474 (d, *J* = 7.3 Hz, 1H), 7.097 (dd, *J* = 9.5 Hz, 1.7 Hz, 2H), 7.019 (d, *J* = 9.5 Hz, 2H), 6.976 (d, *J* = 1.8 Hz, 2H), 3.647 (dd, *J* = 13.6 Hz, 6.6 Hz, 8H), 1.210 (t, *J* = 6.8 Hz, 12H). ^{13}C NMR (126 MHz, DMSO) δ 166.73, 157.54, 155.49, 133.68, 133.13, 131.45, 131.42, 131.31, 130.81, 130.72, 130.66, 114.92, 113.32, 96.32, 45.77, 45.67, 12.90.

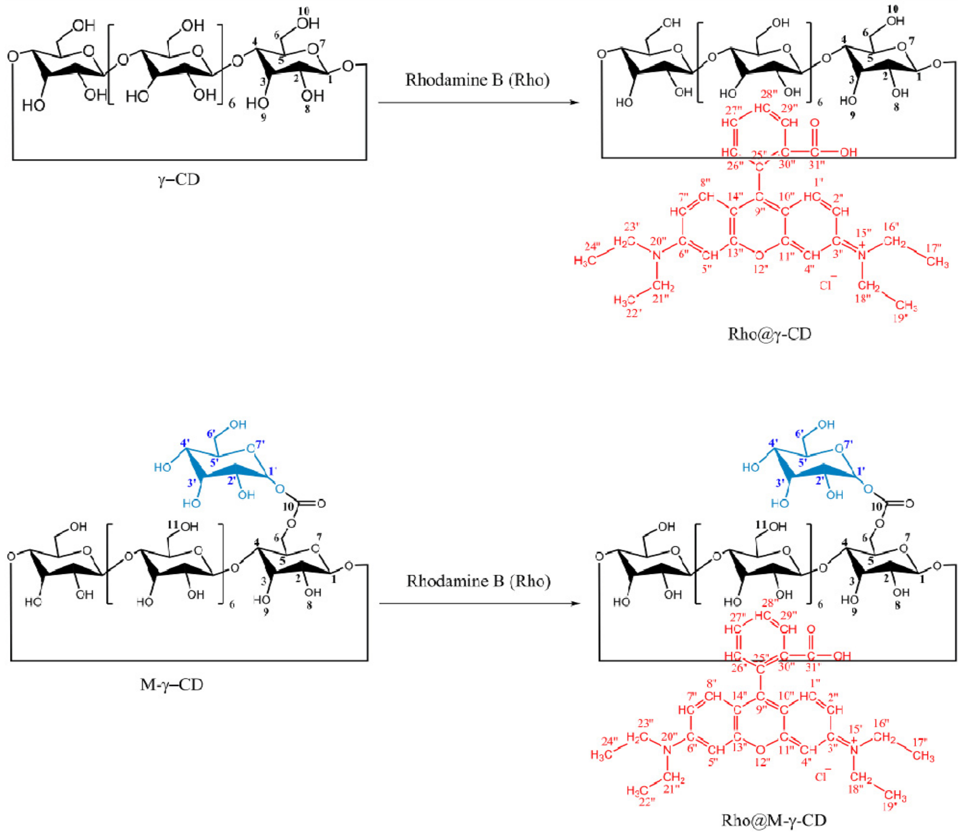

Supplementary Figure 24. Schematic diagram of synthetic route of Rho@γ-CD and Rho@M-γ-CD.

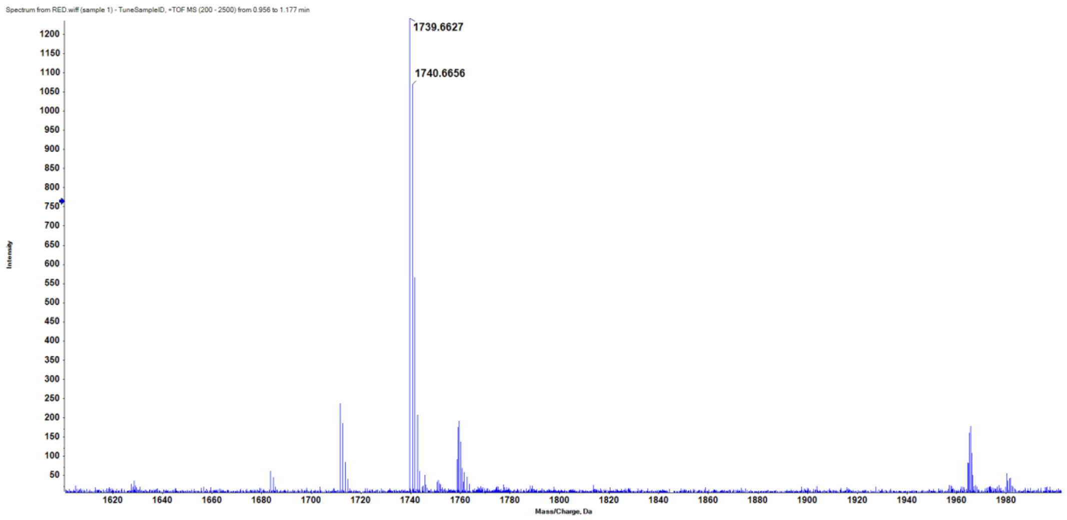

Supplementary Figure 25. ESI-TOF-MS spectrum of Rho@γ-CD. Since inclusion complexes were non-covalent molecular systems, syringe pump with flow rate of 20 L/min was applied in MS analysis to avoid damage to inclusion complexes.

ESI-ToF-MS: calculated for Rho@γ-CD requires *m/z* 1775.6291. Rho@γ-CD, $C_{76}H_{111}ClN_2O_{43}$ [M-Cl]: 12.0000 × 76 + 1.0078 × 111 + 14.0031 × 2 + 15.9949 × 43 = 1740.6605, found *m/z* 1740.6656.

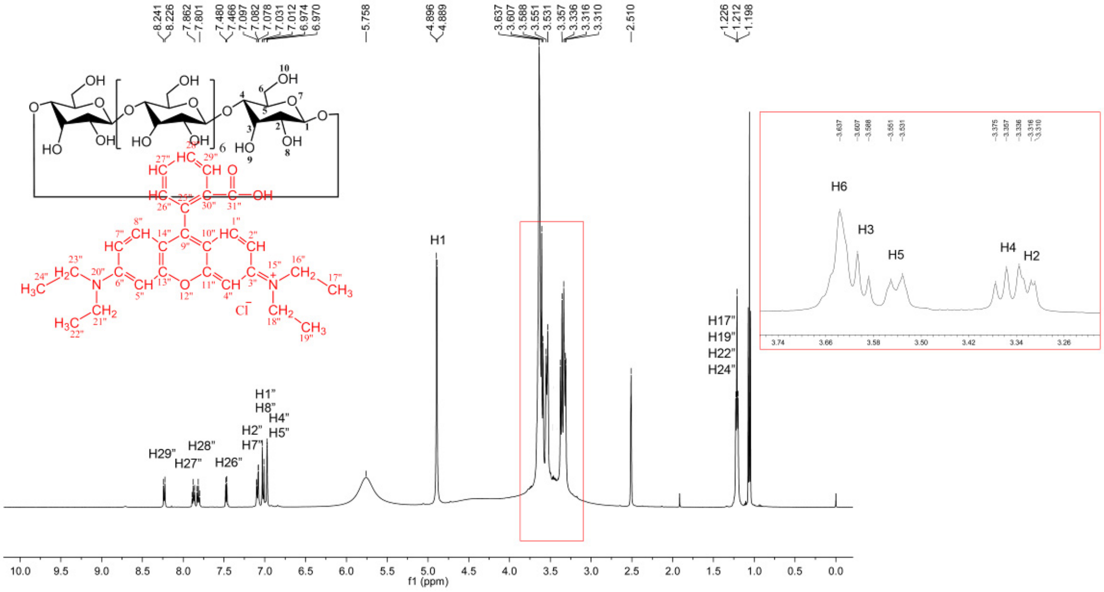

Supplementary Figure 26. ^{1}H NMR spectrum of Rho@γ-CD (500 MHz, DMSO-D6, room temperature).

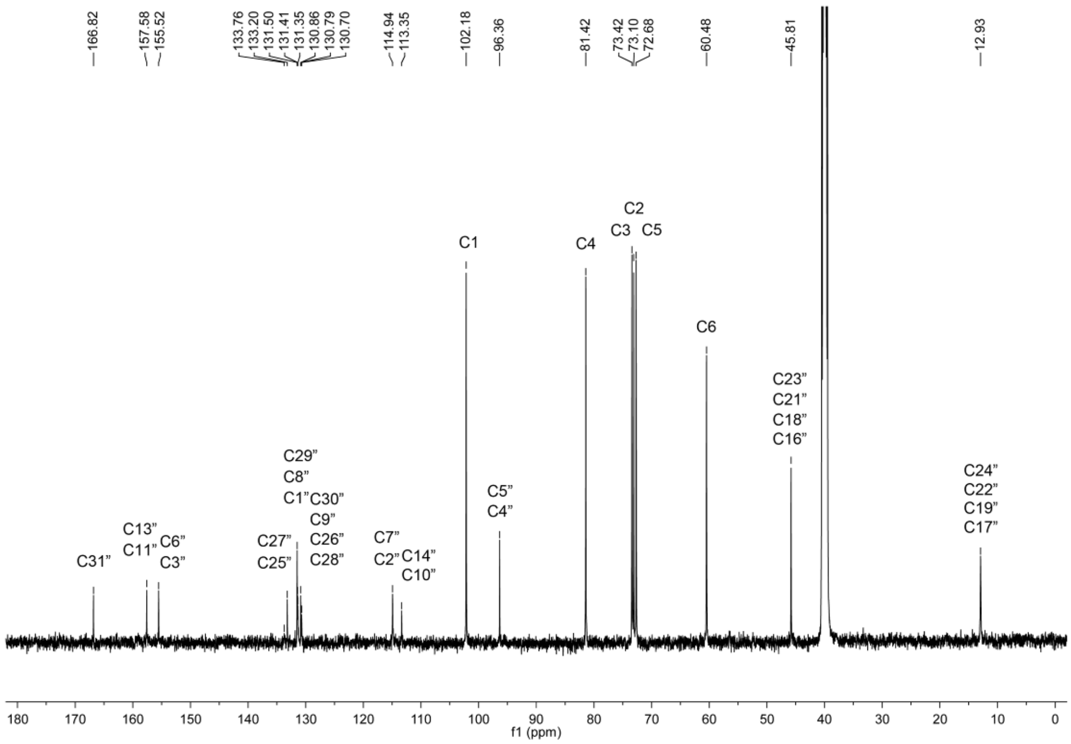

Supplementary Figure 27. ^{13}C NMR spectrum of Rho@γ-CD (500 MHz, DMSO-D6, room temperature).

^{1}H NMR (500 MHz, DMSO-D6) δ 8.234 (d, J = 7.6 Hz), 7.877 (t, J = 7.3 Hz), 7.815 (t, J = 7.3 Hz), 7.473 (d, J = 7.3 Hz), 7.090 (dd, J = 9.5 Hz, 1.6 Hz), 7.021 (d, J = 9.5 Hz), 6.972 (d, J = 1.8 Hz), 4.893 (d, J = 3.1 Hz), 3.637 (s), 3.622 (d, J = 15.0 Hz), 3.597 (d, J = 9.3 Hz), 3.541 (d, J = 9.8 Hz), 3.375-3.336 (m), 3.336-3.310 (m), 1.212 (t, J = 6.9 Hz). ^{13}C NMR (126 MHz, DMSO-D6) δ 166.82, 157.58, 155.52, 133.76, 133.20, 131.50, 131.41, 131.35, 130.86, 130.79, 130.70, 114.94, 113.35, 102.18, 96.36, 81.42, 73.42, 73.10, 72.68, 60.48, 45.81, 12.93.

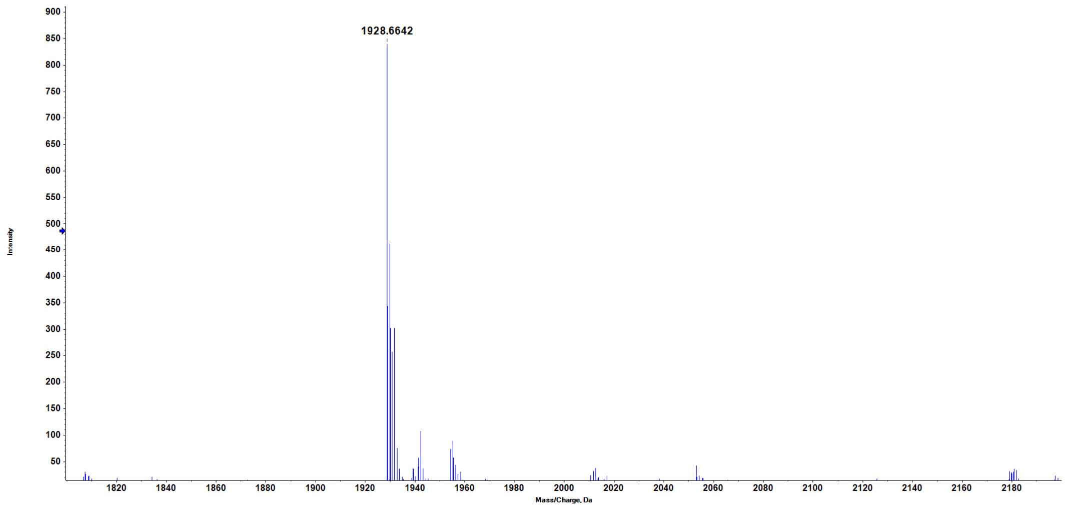

Supplementary Figure 28. ESI-TOF-MS spectrum of Rho@M-γ-CD. Since inclusion complexes were non-covalent molecular systems, syringe pump with flow rate of 20 L/min was applied in MS analysis to avoid damage to inclusion complexes. ESI-ToF-MS: calculated for Rho@M-γ-CD requires *m/z* 1980.6636. Rho@M-γ-CD, $C_{83}H_{121}ClN_2O_{50}$ [M]: 12.0000 × 83 + 1.0078 × 121 + 34.9688 + 14.0030 × 2 + 15.9949 × 50 = 1980.6636, [M-Cl]: 1980.6636 - 34.9688 = 1945.6948. Calculated for $[M\text{-}Cl\text{-}OH]^+$ requires *m/z* 1928.6921, found *m/z* 1928.6642.

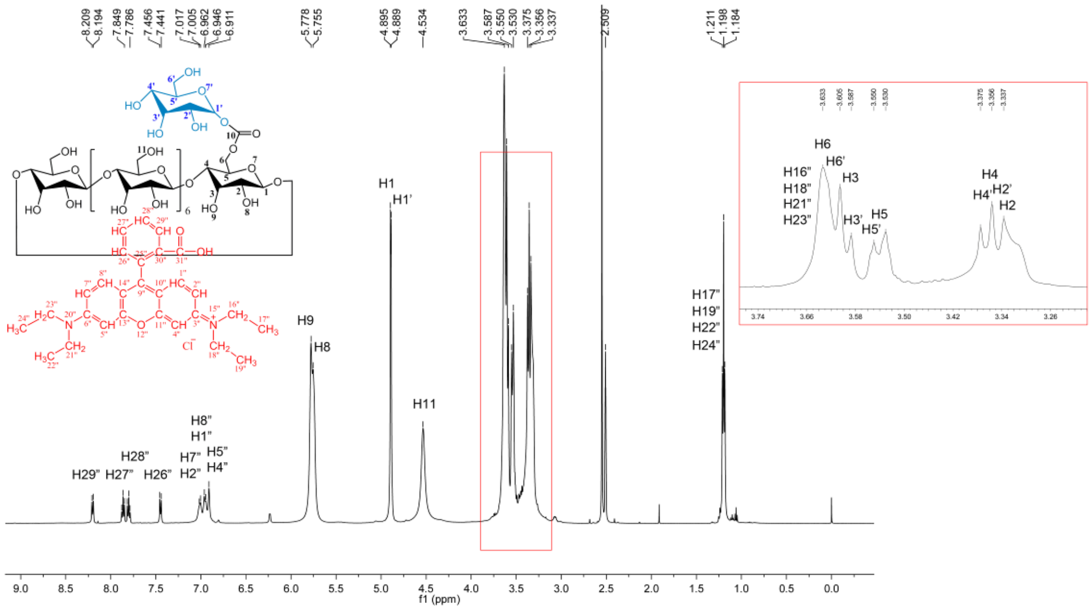

Supplementary Figure 29. ^{13}H NMR spectrum of Rho@M-γ-CD (500 MHz, DMSO-D6, room temperature).

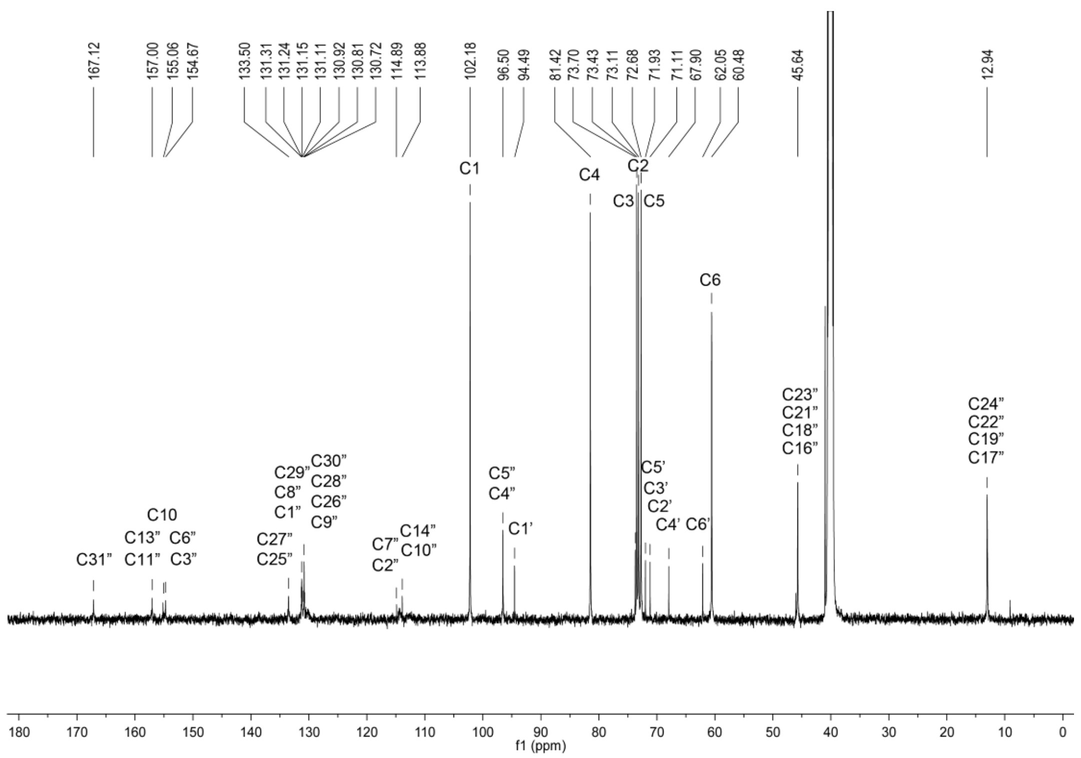

Supplementary Figure 30. ^{13}C NMR spectrum of Rho@M-γ-CD (500 MHz, DMSO-D6, room temperature).

^{1}H NMR (500 MHz, DMSO) δ 8.202 (d, J = 7.7 Hz), 7.864 (t, J = 7.4 Hz), 7.801 (t, J = 7.3 Hz), 7.448 (d, J = 7.4 Hz), 7.011 (d, J = 6.2 Hz), 6.954 (d, J = 8.2 Hz), 6.911 (s), 5.778 (s), 5.755 (s), 4.892 (d, J = 3.3 Hz), 4.889 (s), 4.534 (s), 3.633-3.587 (m) 3.633 (s), 3.619 (d, J = 13.9 Hz), 3.596 (d, J = 9.1 Hz), 3.587 (s), 3.550 (s), 3.540 (d, J = 9.6 Hz), 3.375-3.337 (m), 3.337 (s), 3.365 (d, J = 9.3 Hz), 3.346 (d, J = 9.7 Hz), 3.337 (s), 1.198 (t, J = 6.9 Hz). ^{13}C NMR (126 MHz, DMSO) δ 167.12, 157.00, 155.06, 154.67, 133.50, 131.31, 131.24, 131.15, 131.11, 130.92, 130.81, 130.72, 114.89, 113.88, 102.18, 96.50, 94.49, 81.42, 73.70, 73.43, 73.11, 72.68, 71.93, 71.11, 67.90, 62.05, 60.48, 45.64, 12.94.

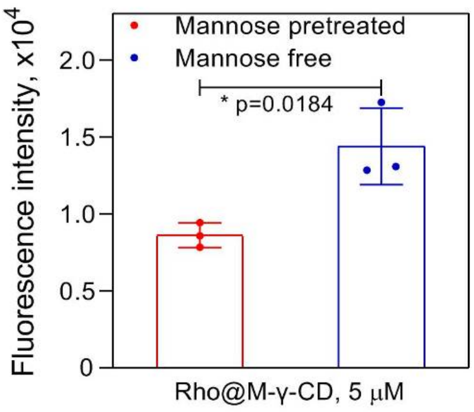

Supplementary Figure 31. The CT26 cells were pre-treated with free mannose for 8 h (0.5 μM) and then co-incubated with Rho@M-γ-CD for 2 h to test whether the high internalization of RG@M-γ-CD toward CRC cells was mediated by the mannose receptor binding. FACS analysis displayed that Rho fluorescence intensity of cells with pre-treatment significantly decreased compared with the cells without pre-treatment. Fluorescence intensity of Rho@M-γ-CD in CT26 cells without or with mannose pre-treatment. N = 3 biological replicates in each group. Data were presented as means ± SD and statistical significance was calculated using two-tailed, unpaired t-test. The exact p values were indicated in the figures.

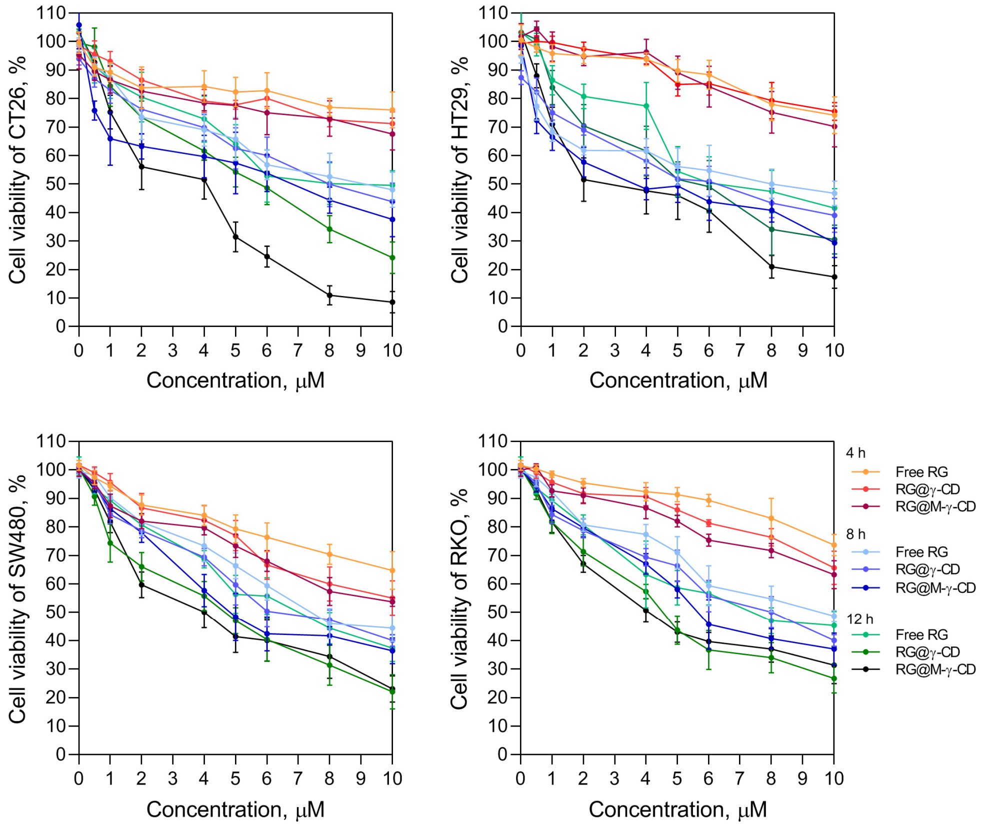

Supplementary Figure 32. Cytotoxicity test in CT26, HT29, SW480 and RKO cells

treated with RG, RG@γ-CD and RG@M-γ-CD for 4 h, 8 h and 12 h. Concentrations of equivalent RG ranged from 0.5 μM to 10 μM. RG, RG@γ-CD and RG@M-γ-CD showed weak cytotoxicity on CRC cells within 4 h. As a multi-kinase inhibitor, RG primarily targeted the protein kinases involved in the signaling of tyrosine kinases, oncogenesis and tumor microenvironment, rather than inducing direct cytotoxicity. The treatment was thus prolonged to 8 h or 12 h, and the inhibitory effects gradually increased. N = 5 biological replicates in each group. Data were expressed as means ± SD.

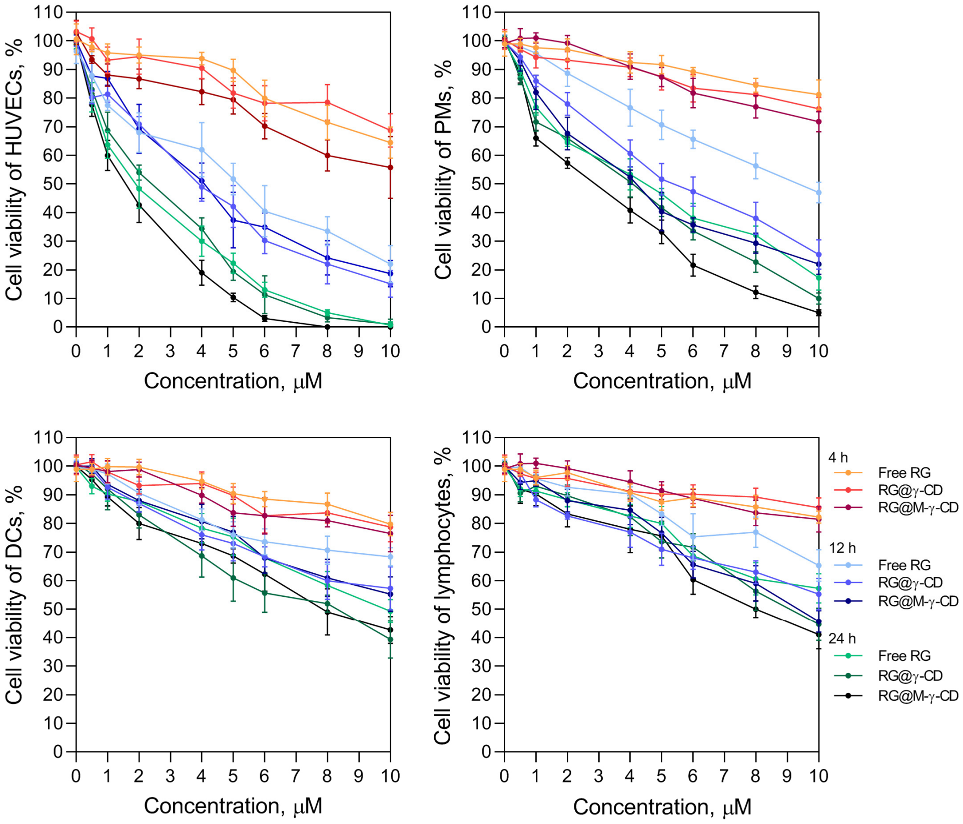

Supplementary Figure 33. Cytotoxicity test in HUVECs, peritoneal macrophages (PMs), dendritic cells (DCs) and splenocytes treated with RG, RG@M-γ-CD and RG@M-γ-CD for 4 h, 12 h and 24 h. Concentrations of equivalent RG ranged from 0.5 μM to 10 μM. Within 4 h, RG, RG@γ-CD and RG@M-γ-CD induced negligible cell death, which was consistent with the MTT result of CRC cells. When the treatment prolonging to 12 h or 24 h, cell viability dropped in a dosage-dependent manner. N = 3 biological replicates in each group. Data were expressed as means ± SD.

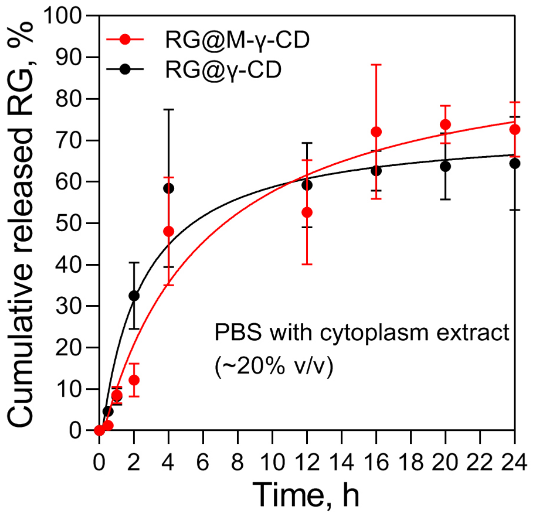

Supplementary Figure 34. Drug release kinetics of RG@γ-CD and RG@M-γ-CD CNPs in PBS with cytoplasm extract (~20% v/v in PBS). N = 3 independent samples in each group and data were expressed as means ± SD. Cytoplasm was separated using Nuclear/Cytosol Fractionation Kit (Biovision) according to the manufacturer's instructions. Briefly, the cells in culture dish were placed on ice and were washed with cold PBS. PBS-EDTA solution (1.0 mL) was added into the culture dish. After co-incubation with PBS-EDTA for 10 min, cells were harvested and centrifuged at 3,000 rpm for 5 min at 4 °C. Then the cells were resuspend with H buffer and incubated on ice. The supernatant was centrifuged at 14,000 rpm for 10 min, and the obtained supernatant contained cytoplasmic contents. RG@γ-CD and RG@M-γ-CD CNPs were incubated with cytoplasm extract (~20% v/v in PBS) and the released RG was monitored by HPLC. Within co-incubation with cytoplasm extract for 24 h, 63.5 ± 13.2% and 76.4 ± 6.7% of RG was released from RG@γ-CD and RG@M-γ-CD, respectively. The sustained release property was attributed to the stepwise dissociation of CNPs driven by the cytoplasm extract.

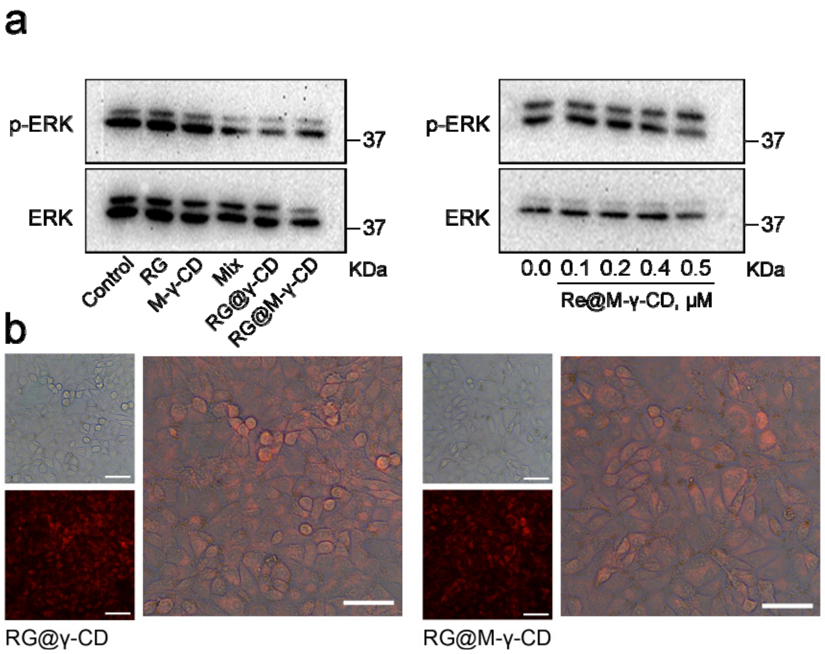

Supplementary Figure 35. (**a**) Phosphorylation of ERK in HT29 cells treated with RG, M-γ-CD, Mix, RG@γ-CD and RG@M-γ-CD (equivalent RG, 0.5 μM). HT29 cells were treated with RG, M-γ-CD, Mix, RG@γ-CD and RG@M-γ-CD for 6 h. Then the stimuli were removed and the cells were cultured in serum media for 6 h. Afterwards, the inhibitory effect of RG@M-γ-CD CNPs on phosphorylation of ERK was evaluated by western blot. Dose-dependent inhibition of phosphorylation of ERK in HT29 cells treated with RG@M-γ-CD at various concentrations (0.1-0.5 μM). N = 3 biological replicates in each group. (**b**) The internalization of Rho@γ-CD and Rho@ M-γ-CD upon HUVECs with 2 h treatment (equivalent Rho, 5.0 μM). The cell internalization was observed using inverted fluorescence microscopy. Scale bar, 50 μm. N = 3 biological replicates in each group. Both CNPs exhibited similar internalization efficiency within 2 h of incubation, implying that the CNPs could be taken up by HUVECs and confirming that the M-γ-CD was specific strategy for targeting CRC cells and colonic macrophages.

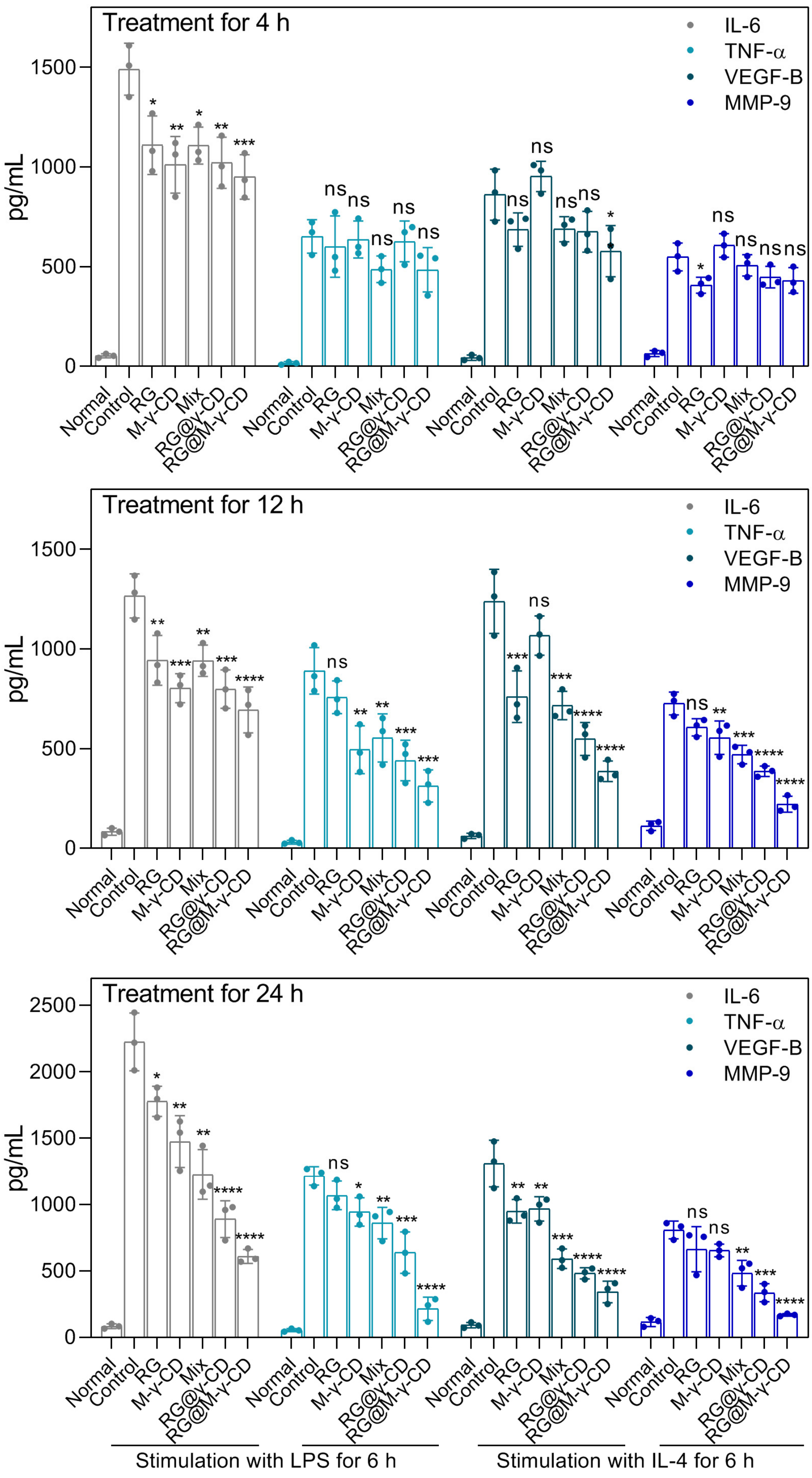

Supplementary Figure 36. The regulatory effect of RG@M-γ-CD on the polarization of peritoneal macrophages. PMs were subjected to the treatments for 4 h, 12 h and 24 h (equivalent RG, 0.5 μM), then were incubated with LPS or IL-4 for 6 h to induce polarization. The protein expression levels of phenotype-related cytokines were

determined by mouse ELISA kit (Neo bioscience) according to the manufacturer's instructions, including pro-inflammation cytokines (IL-6 and TNF-α) and M2-related factors (VEGF-B and MMP-9). N = 3 biological replicates in each group. Data were presented as means ± SD. Statistical significance was calculated using one-way ANOVA followed by Dunnett’s multiple comparison test (*$p \leq 0.05$, **$p \leq 0.01$, ***$p \leq 0.001$, ****$p \leq 0.0001$, treatment group vs. control, the exact p values were indicated in Source Date).

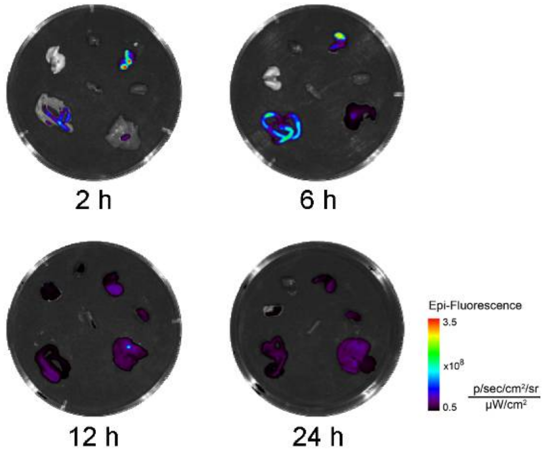

Supplementary Figure 37. Representative ex vivo fluorescence imaging of main organs at 2 h, 6 h, 12 h and 24 h post administration of Rho (10 μg/g). N = 3 biological replicates in each group. Supplementary Figure 37 refers to Figure 5b.

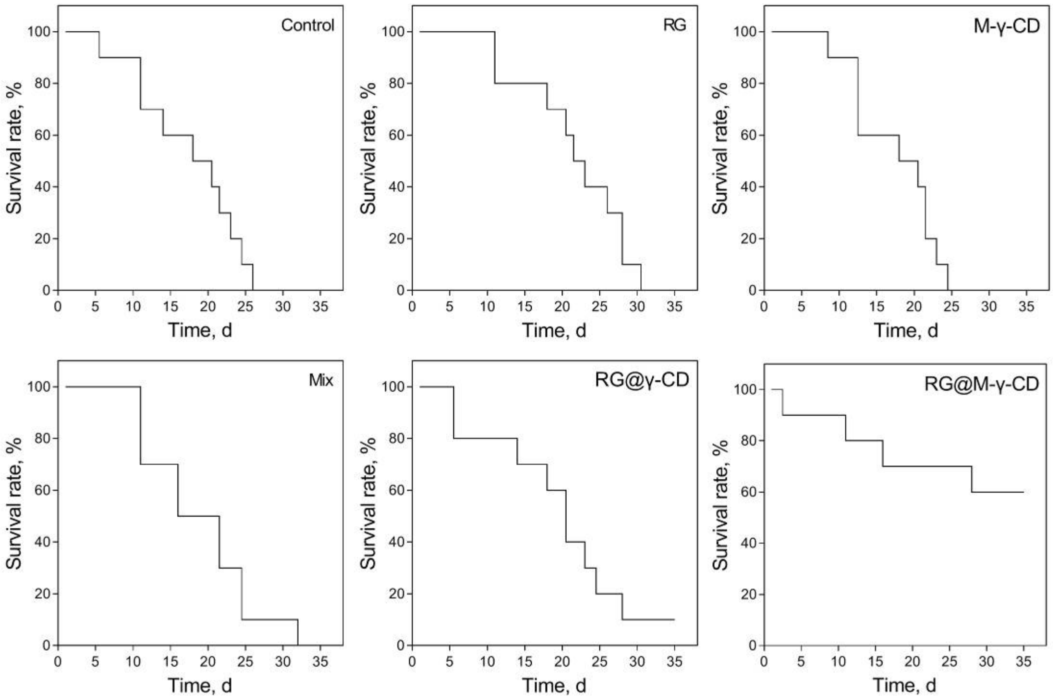

Supplementary Figure 38. Kaplan-Meier survival analysis for CAC mice under different treatments (n=10). Supplementary Figure 38 refers to Figure 5g-5j.

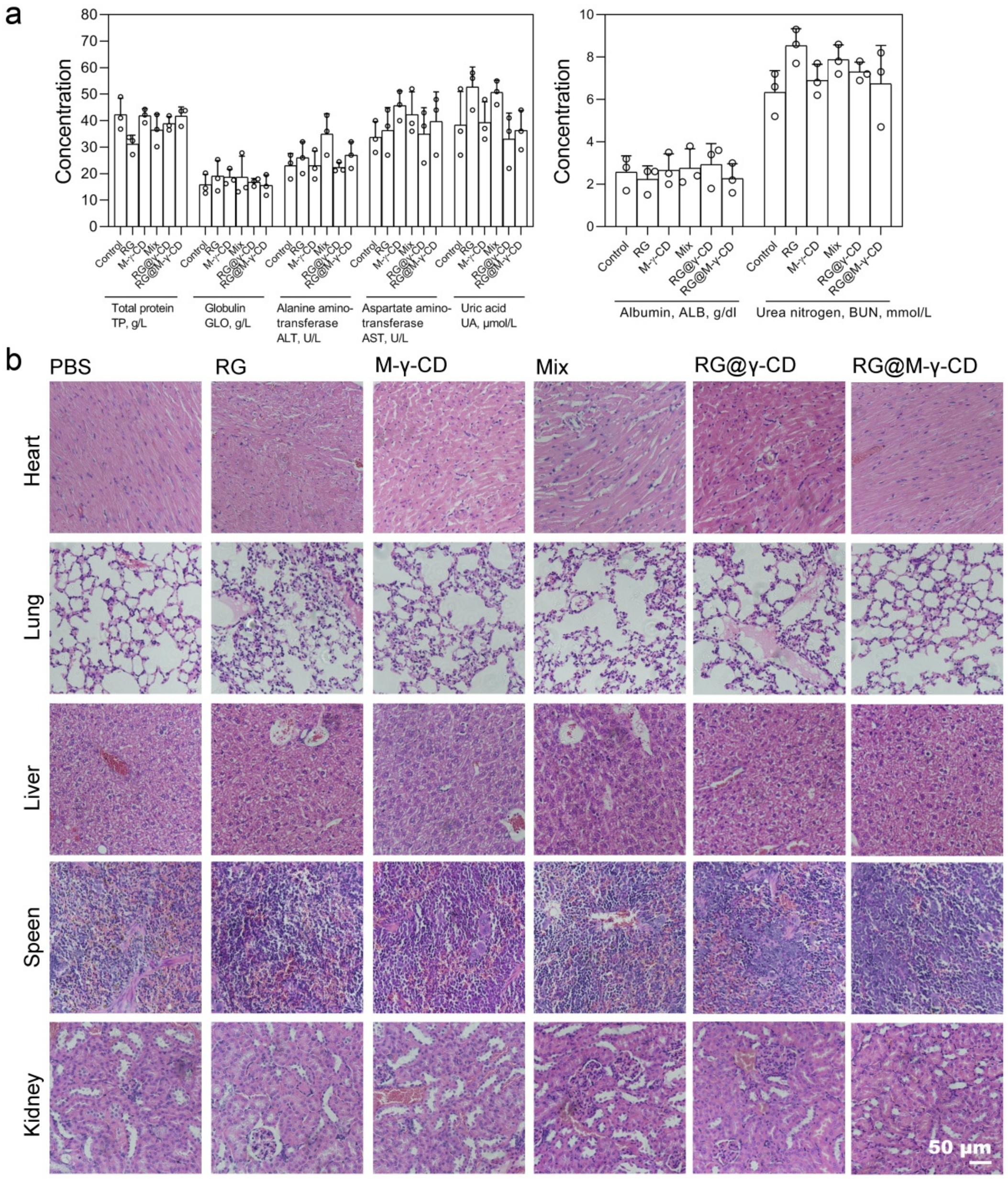

Supplementary Figure 39. In vivo safety test. (**a**) The healthy mice were orally administrated with different formulations at drug dosage of 10 μg/g for three times. At day 21 after administration, blood biochemistry measurement was conducted. The RG@M-γ-CD CNPs-treated mice showed similar parameters of biochemical markers associated to liver and kidney functions, as compared to the control mice. While, administration with RG or mix resulted in dysregulations in some parameters, including alanine aminotransferase and aspartate aminotransferas. N = 3 biological replicates in each group. Data were presented as means ± SD. (**b**) Representative images of H&E staining of heart, liver, spleen, lung and kidney from each treatment group. Scale bar, 50 μm. Images were representative of 3 biologically independent mice in each group.

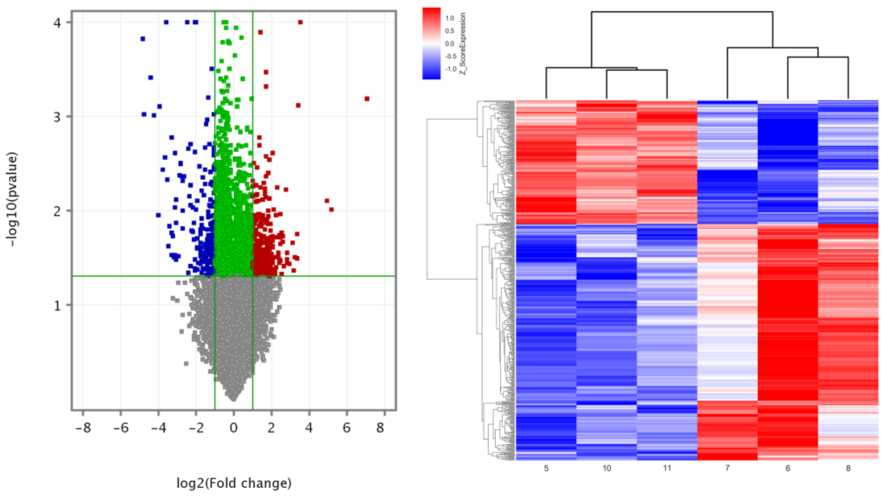

Supplementary Figure 40. The mRNA analysis was performed on Control group and RG@M-γ-CD gourp to investigate the molecular mechanism that RG@M-γ-CD exerted mediations on colon tumor and tumor microenvironment. Total mRNAs of colon tissues with tumors were quantified by Nano Drop ND-2000 (Thermo Fisher Scientific) and the mRNA integrity was verifed using Agilent Bioanalyzer 2100 (Agilent Technologies). The total mRNAs were transcribed into double strand cDNAs, then the cDNA was labeled with Cyanine-3-CTP. The labeled cRNAs were hybridized onto Agilent Sure Print G3 Mouse GE V2.0 microarray (8*60K, Design ID:074809). After washing, the microarray was scanned with Agilent Scanner G2505C (Agilent Technologies). Data acquisition and array image analysis were performed using Feature Extraction software (version10.7.1.1, Agilent Technologies) and Gene spring (version13.1, Agilent Technologies) to quantify the mRNA expression levels. Differentially expressed mRNAs were then identified through fold changes and the corresponding statistical significances were calculated using unpaired two-tailed t-test. Fold change ≥ 2.0 with p value ≤ 0.05 was used as threshold set to determine the up- and down-regulations. n = 3 biologically independent mice in each group. Volcano plot of the filtered differential data based on fold-change and p value. Heatmap of differential expression genes clustered by distance-marking method. Color bar indicated Z Scores.

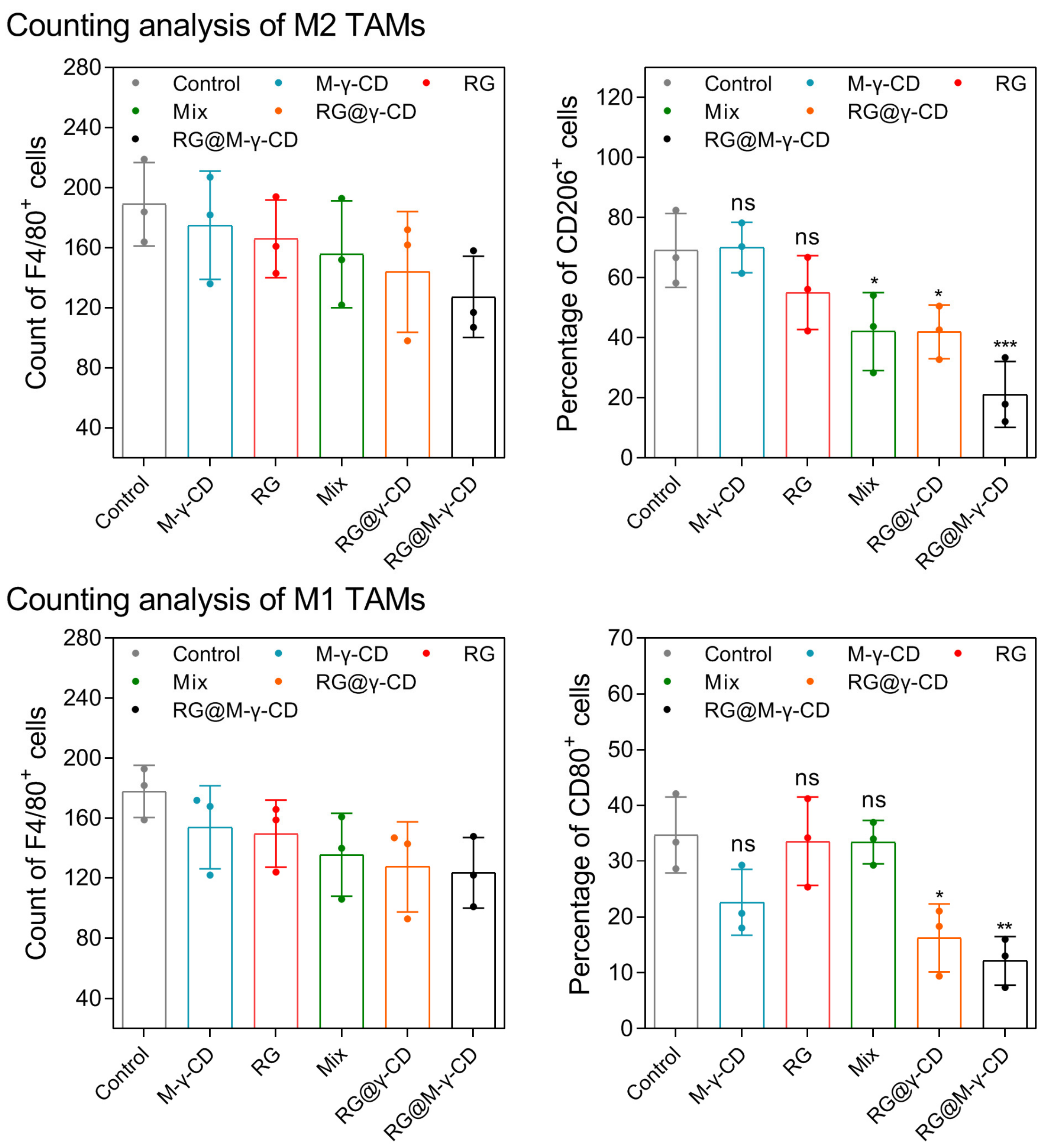

Supplementary Figure 41. Immunofluorescence analysis was performed to demonstrate the TAM deactivation effect of RG@M-γ-CD CNPs. The colon tumor tissues from treatment groups were sectioned and stained with F4/80, CD206 and CD80 antibodies. The F4/80$^+$ cells were counted for the treatment groups and the percentage of CD206$^+$ (or CD80$^+$) cells in F4/80$^+$ cells was calculated to demonstrate the intratumoral TAM infiltration. Counting analysis of intratumoral TAMs based on immunofluorescence data. Above: Cell count of F4/80$^+$ cells in 150 μm × 150 μm regions of immunofluorescence images from the treatment groups, and the percentage of CD206$^+$ cells in F4/80$^+$ cells (M2). Below: Cell count of F4/80$^+$ cells in 150 μm × 150 μm regions of immunofluorescence images from the treatment groups, and the percentage of CD80$^+$ cells in F4/80$^+$ cells (M1). N = 3 biological replicates in each

group. Data were presented as means ± SD. Statistical significance was calculated using one-way ANOVA followed by Dunnett's multiple comparison test (*p ≤ 0.05, **p ≤ 0.01, ***p ≤ 0.001, ****p ≤ 0.0001, treatment group vs. control, the exact p values were indicated in Source Date). Supplementary Figure 41 refers to Figure 6f.

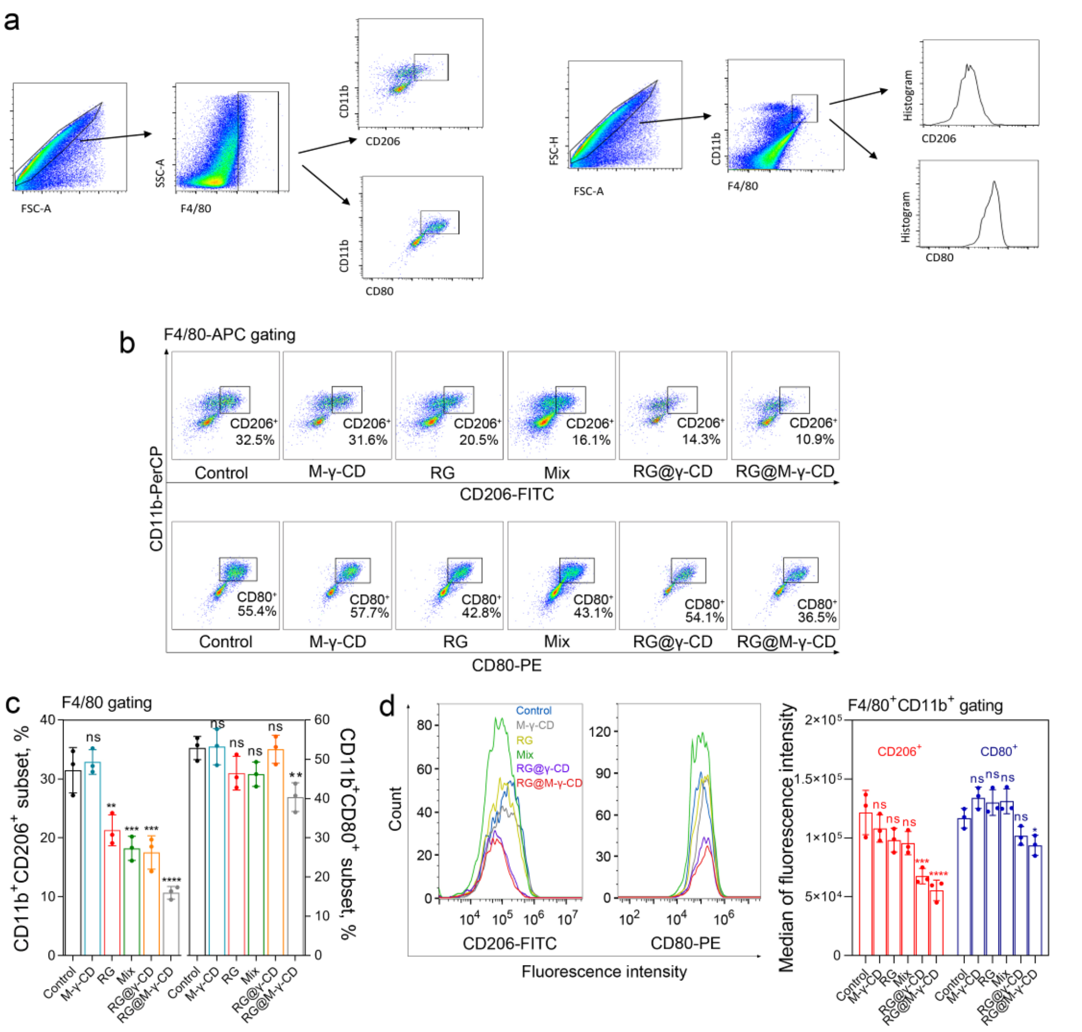

Supplementary Figure 42. FACS analysis using colon lamina propria cells of tumor regions for CAC mouse model. The colon lamina propria cells were separated from tumor regions and FACS analysis was performed to quantify the subsets of $CD206^+$ and $CD80^+$ macrophages in $F4/80^+$ $CD11b^+$ population. (**a**) FACS sequential gating strategy. (**b**) Representative contour plots of $CD11b^+$ $CD206^+$ and $CD11b^+$ $CD80^+$ subsets gated on the $F4/80^+$ cell set for different treatment groups. (**c**) Statistical analysis of TAM (M1, M2) polarization. N = 3 biological replicates in each group. Data were presented as means ± SD. (**d**) Representative fluorescence intensities and statistical analysis of $CD206^+$ and $CD80^+$ subsets in macrophage subset for different

treatment groups. N = 3 biological replicates in each group. Data were presented as means ± SD. Statistical significance for **c** and **d** was calculated using one-way ANOVA followed by Dunnett's multiple comparison test (*$p \leq 0.05$, **$p \leq 0.01$, ***$p \leq 0.001$, ****$p \leq 0.0001$, treatment group vs. control, the exact p values were indicated in Source Date).

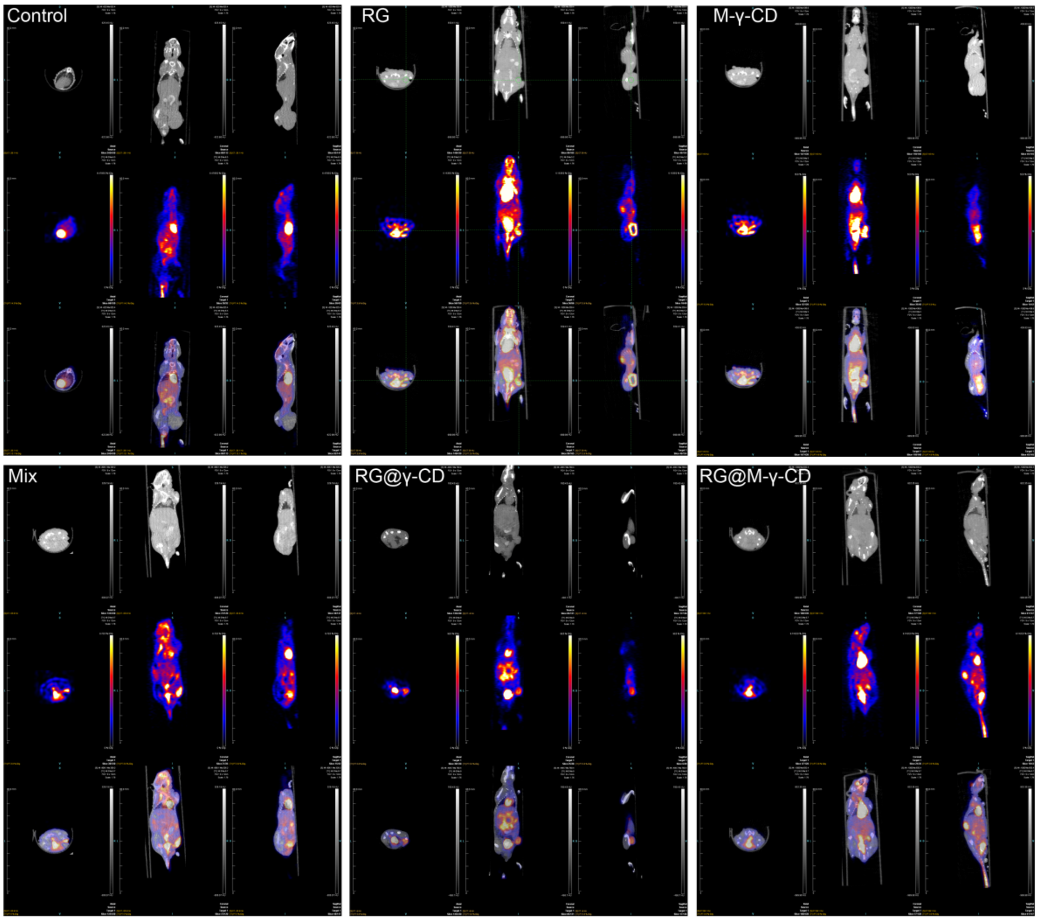

Supplementary Figure 43. Micro-PET/CT image of the CT26 tumor-bearing mice from different treatment groups (PBS, M-γ-CD, RG, Mix, RG@γ-CD and RG@M-γ-CD CNPs). Images were representative of 3 biologically independent mice in each group. Supplementary Figure 43 refers to Figure 7d.

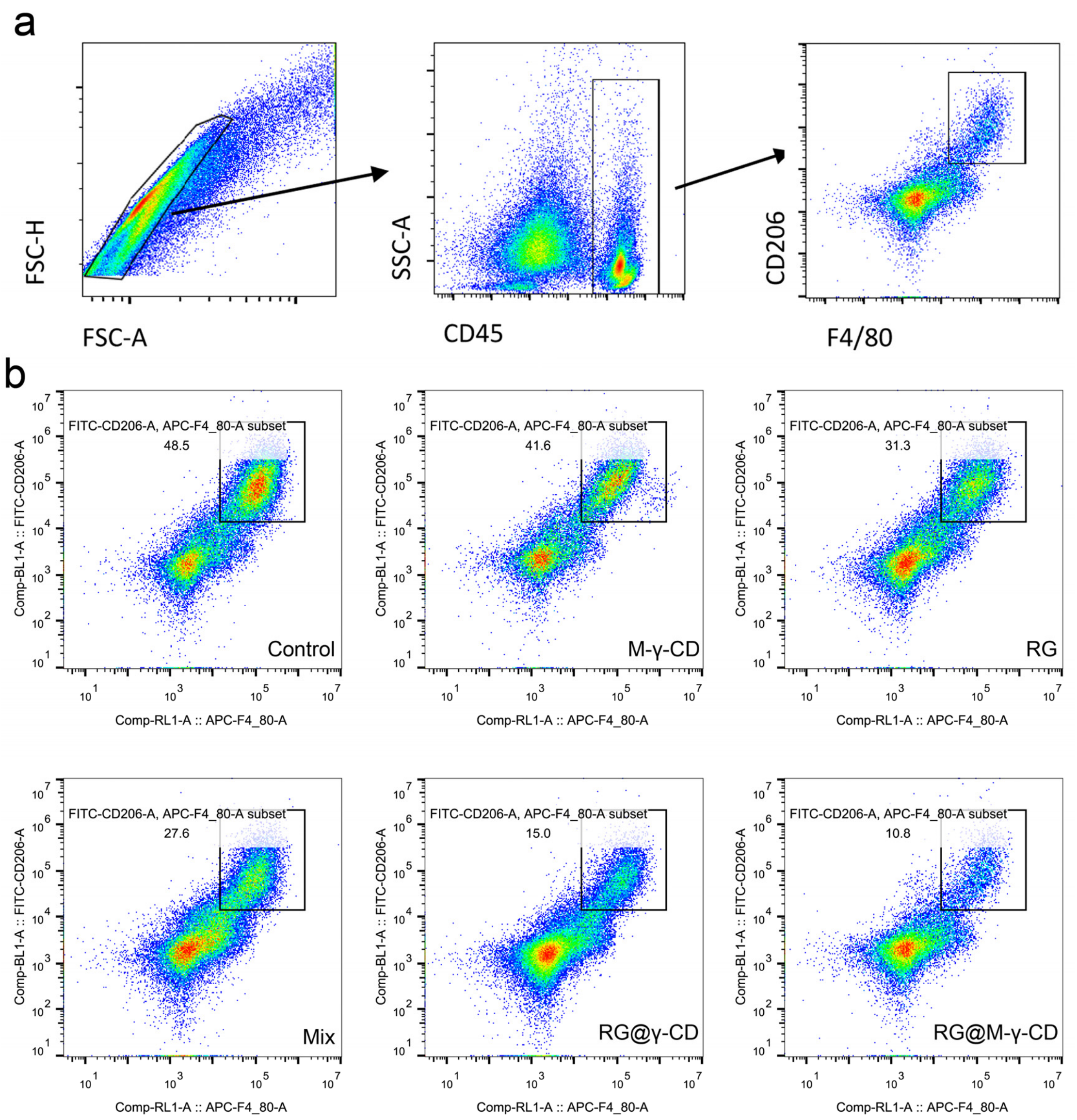

Supplementary Figure 44. FACS analysis of TAMs within tumor tissues from different treatment groups for CT26 model. (a) FACS sequential gating strategy. (b) Representative contour plots of $CD206^+$ $F4/80^+$ subsets gated on $CD45^+$ cell set in CT26 tumor tissues after different treatments. N = 3 biological replicates in each group. FACS analysis showed a distinct reduction of TAM infiltration in the RG@M-γ-CD group as compared to that in control group. Supplementary Figure 44 refers to Figure 7i.

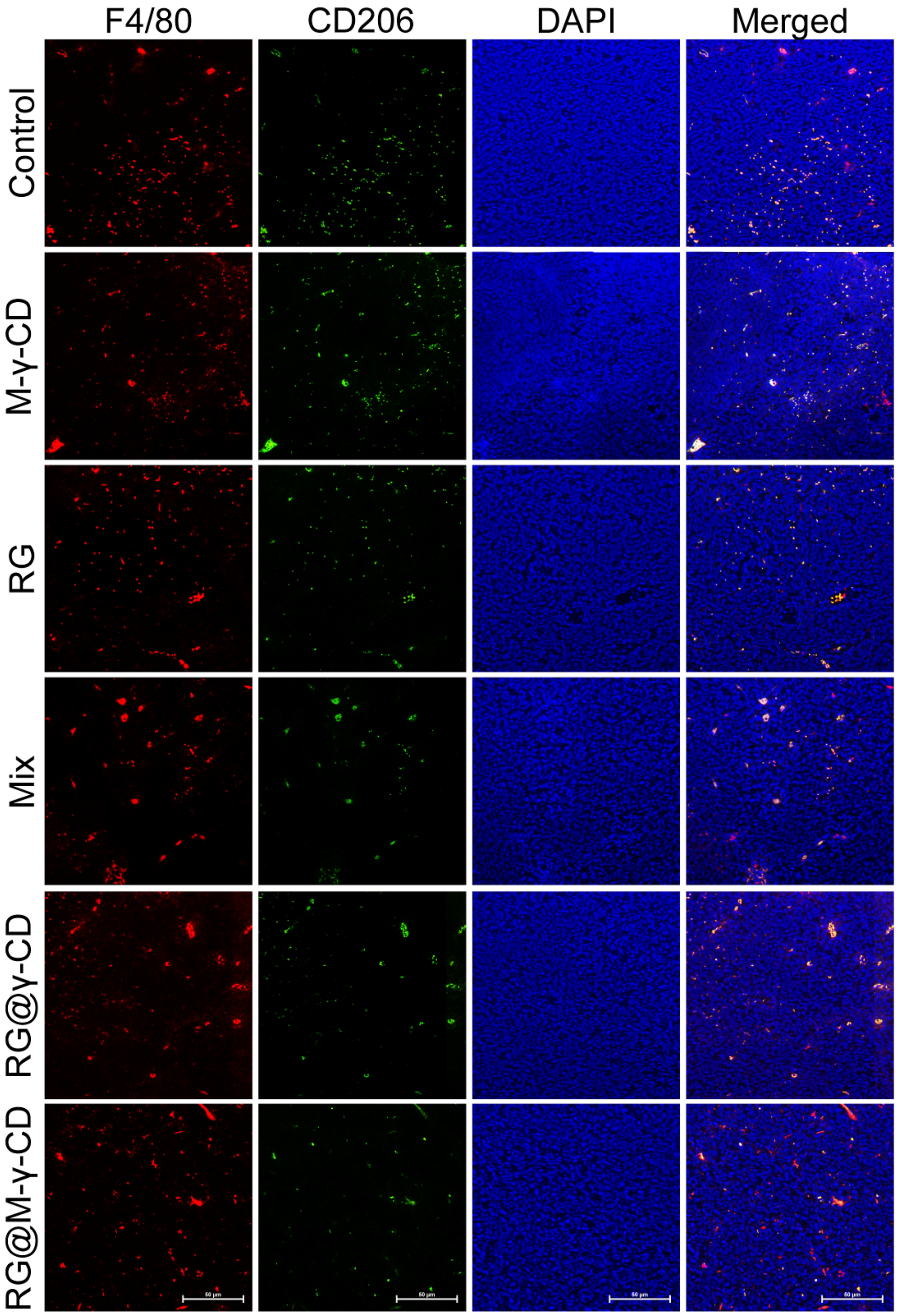

Supplementary Figure 45. Immunofluorescence analysis on CT26 tumor sections by F4/80 and CD206 staining to detect the intratumoral TAMs. Representative images of tumor areas from treatment groups were captured using inverted fluorescence microscopy. Scale bar, 50 μm. Images were representative of 3 biologically independent mice in each group.

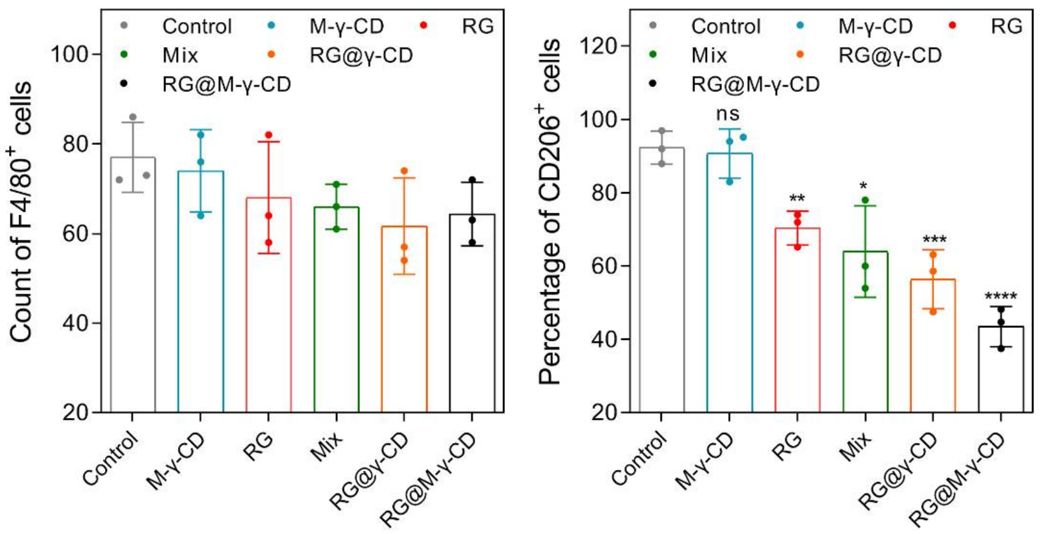

Supplementary Figure 46. Counting analysis of intratumoral TAMs based on immunofluorescence data. Cell count of F4/80$^+$ cells in 150 μm × 150 μm regions of immunofluorescence images from the treatment groups, and the percentage of CD206$^+$ cells in F4/80$^+$ cells. N = 3 biological replicates in each group. Data were presented as means ± SD. Statistical significance was calculated using one-way ANOVA followed by Dunnett's multiple comparison test (*$p \le 0.05$, **$p \le 0.01$, ***$p \le 0.001$, ****$p \le 0.0001$, treatment group vs. control, the exact p values were indicated in Source Date). RG@M-γ-CD treatment resulted in the decreased F4/80$^+$ CD206$^+$ cells compared with other groups, indicating the effective TAM deactivation. Supplementary Figure 46 refers to Supplementary Figure 45.

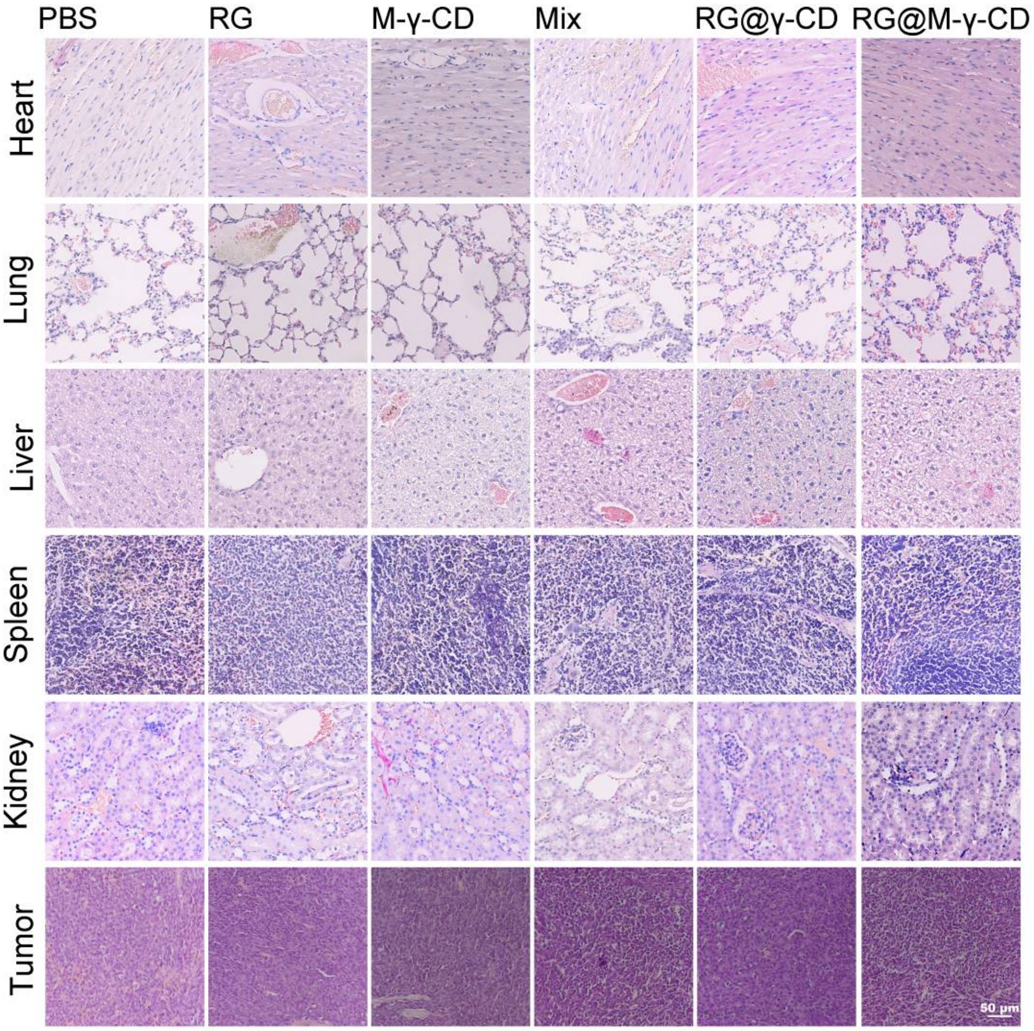

Supplementary Figure 47. Representative images of H&E staining of heart, liver, spleen, lung, kidney and tumor from CT26 tumor-bearing mice in each treatment group. Scale bar, 50 μm. Images were representative of 3 biologically independent mice in each group.

Supplementary Table 1. ^{13}C NMR and ^{1}H NMR chemical shifts of Mannose in DMSO-D6. Supplementary Table 1 refers to Supplementary Figure 1, 2.

Carbon No.	^{13}C δ (ppm)	HSQC ^{1}H δ (ppm)	*J* value
C1	94.51	4.872 (H1)	dd, *J* = 4.3 Hz, 0.8 Hz
C2	71.94	3.526 (H2)	d, *J* = 3.6 Hz
C3	71.12	3.517 (H3)	dd, *J* = 9.3 Hz, 3.1 Hz
C4	67.90	3.355 (H4)	td, *J* = 9.1 Hz, 5.5 Hz
C5	73.72	3.510-3.486 (H5)	m
C6	62.06	3.628 (H6)<br>3.456-3.422 (H6)	ddd, *J*= 11.1 Hz, 5.1Hz, 1.7 Hz<br>m
Oxygen No.		^{1}H δ (ppm)	*J* value
O8	/	6.224 (H8)	d, *J* = 4.4 Hz
O9	/	4.495 (H9)	d, *J* = 4.1 Hz
O10	/	4.421 (H10)	d, *J* = 5.7 Hz
O11	/	4.607 (H11)	d, *J* = 5.5 Hz
O12	/	4.316 (H12)	d, *J* = 5.7 Hz

Supplementary Table 2. ^{13}C NMR and ^{1}H NMR chemical shifts of γ-CD in DMSO-D6. Supplementary Table 2 refers to Supplementary Figure 3, 4.

Carbon No.	^{13}C δ (ppm)	HSQC ^{1}H δ (ppm)	*J* value
C1	102.19	4.888 (H1)	d, *J* = 3.5 Hz
C2	73.09	3.334-3.297 (H2)	m
C3	73.42	3.589 (H3)	dd, *J* = 9.3 Hz, 2.2 Hz
C4	81.43	3.371-3.353 (H4)	m
C5	72.69	3.533 (H5)	d, *J* = 9.8 Hz
C6	60.48	3.625 (H6)	d, *J* = 4.6 Hz
Oxygen No.		^{1}H δ (ppm)	*J* value
O8	/	5.750 (H8)	d, *J* = 7.0 Hz
O9	/	5.773 (H9)	d, *J* = 2.5 Hz
O10	/	4.531 (H10)	t, *J* = 5.6 Hz

Supplementary Table 3. ^{13}C NMR and ^{1}H NMR chemical shifts of M-γ-CD in DMSO-D6 and the chemical shift comparison between α-/β-anomers of mannose and M-γ-CD in D_2O. Supplementary Table 3 refers to Supplementary Figure 7-10.

Carbon No.	^{13}C δ (ppm)	HSQC ^{1}H δ (ppm)	*J* value
C1	102.19	4.887 (H1)	d, *J* = 3.5 Hz
C2	73.10	3.325 (H2)	d, *J* = 8.1 Hz
C3	73.42	3.588 (H3)	d, *J* = 9.6 Hz
C4	81.43	3.370-3.333 (H4)	m
C5	72.68	3.533 (H5)	d, *J* = 9.8 Hz
C6	60.48	3.625 (H6)	d, *J* = 4.3 Hz
C10	154.22	/	/
C1'	94.50	4.884 (H1')	s
C2'	71.93	3.333 (H2')	s
C3'	71.10	3.578 (H3')	s
C4'	67.89	3.370-3.359 (H4')	m
C5'	73.70	3.543 (H5')	s
C6'	62.05	3.620 (H6')	s
Oxygen No.		^{1}H δ (ppm)	*J* value
O8	/	5.758 (H8)	d, *J* = 6.4 Hz
O9	/	5.781 (H9)	d, *J* = 1.7 Hz
O11	/	4.531 (H11)	t, *J* = 5.6 Hz
^{1}H NMR chemical shifts comparison between α-/β-anomers of mannose and M-γ-CD in D_2O			
	α-anomer	β-anomer	M-γ-CD
H1	5.05	4.77	5.06
H4	3.52	3.45	3.55

Supplementary Table 4. ^{13}C NMR and ^{1}H NMR chemical shifts of RG in DMSO-D6. Supplementary Table 4 refers to Supplementary Figure 11, 12.

Carbon No.	^{13}C δ (ppm)	HSQC ^{1}H δ (ppm)	*J* value
C1	153.03	/	/
C2	109.51-109.42	7.435 (H2)	d, *J* = 2.6 Hz
C3	165.98	/	/
C4	114.64	7.190 (H4)	dd, *J* = 5.6 Hz, 2.6 Hz
C5	150.98	8.536 (H5)	d, *J* = 5.6 Hz
C7	164.21	/	/
C9	26.51	2.806 (H9)	d, *J* = 4.9 Hz
C11	148.66-148.58	/	/
C12	109.69	7.343 (H12)	dd, *J*= 11.6Hz, 2.7 Hz
C13	154.23	/	/
C14	125.45-125.37	/	/
C15	123.11	8.175 (H15)	t, *J* = 9.1 Hz
C16	117.61-117.60	7.080 (H16)	dd, *J* = 8.9 Hz, 1.7 Hz
C18	152.66-152.28	/	/
C20	139.51	/	/
C21	117.19-117.06	8.135 (H21)	s
C22	127.67-126.94	/	/
C23	126.54	/	/
C24	124.36-123.46	7.630 (H24)	s
C25	132.60	7.633 (H25)	s
C26	123.04-122.19	/	/
Nitrogen No.		^{1}H δ (ppm)	*J* value
N8	/	8.796 (H8)	q, *J* = 4.6 Hz
N17	/	8.743 (H17)	d, *J* = 1.6 Hz
N19	/	9.529 (H19)	s

Supplementary Table 5. ^{13}C NMR and ^{1}H NMR chemical shifts of RG@M-γ-CD in DMSO-D6. Supplementary Table 5 refers to Supplementary Figure 14, 15.

Carbon No.	^{13}C δ (ppm)	HSQC ^{1}H δ (ppm)	*J* value
C1	102.20	4.896 (H1)	d, *J* = 3.6 Hz
C2	73.10	3.319 (H2)	dd, *J* = 9.6 Hz, 3.2 Hz
C3	73.43	3.598 (H3)	dd, *J* = 9.4 Hz, 1.8 Hz
C4	81.44	3.380-3.342 (H4)	m
C5	72.69	3.543 (H5)	d, *J* = 9.8 Hz
C6	60.49	3.634 (H6)	d, *J* = 4.6 Hz
C10	152.37	/	/
C1'	94.51	4.892 (H1')	s
C2'	71.94	3.342-3.326 (H2')	m
C3'	71.11	3.589 (H3')	d, *J* = 1.9 Hz
C4'	67.91	3.369 (H4')	d, *J* = 10.3 Hz
C5'	73.70	3.553 (H5')	s
C6'	62.06	3.630 (H6')	s
C1"	153.05	/	/
C2"	109.45	7.426 (H2")	d, *J* = 2.6 Hz
C3"	166.00	/	/
C4"	114.68	7.190 (H4")	dd, *J* = 5.6 Hz, 2.6 Hz
C5"	151.03	8.534 (H5")	d, *J* = 5.6 Hz
C7"	164.24	/	/
C9"	26.54	2.800 (H9")	d, *J* = 4.8 Hz
C11"	148.73-148.65		/
C12"	109.72-109.55	7.340 (H12")	dd, *J* = 11.6 Hz, 2.7 Hz
C13"	154.32	/	/
C14"	125.45-125.37	/	/
C15"	123.18-123.13	8.164 (H15")	d, *J* = 9.0 Hz
C16"	117.63-117.61	7.089-7.066 (H16")	m
C18"	152.72	/	/
C20"	139.55	/	/
C21"	117.19-117.14	8.135 (H21")	d, *J* = 1.1 Hz
C22"	127.68-126.95	/	/
C23"	126.56	/	/
C24"	123.52	7.634 (H24")	s
C25"	132.63	7.634 (H25")	s
C26"	122.22	/	/
Oxygen No.	δ (ppm)	^{1}H δ (ppm)	*J* Vaule
O8	/	5.751 (H8)	d, *J* = 7.0 Hz
O9	/	5.776 (H9)	d, *J* = 2.3 Hz,
O11	/	4.530 (H11)	t, *J* = 5.6 Hz
Nitrogen No.	δ (ppm)	^{1}H δ (ppm)	*J* Vaule
N8"	/	8.795 (H8")	d, *J* = 4.6 Hz
N17"	/	8.777 (H17")	d, *J* = 3.6 Hz
N19"	/	9.566 (H19")	s

Supplementary Table 6. ^{13}C NMR and ^{1}H NMR chemical shifts of RG@γ-CD in DMSO-D6. Supplementary Table 6 refers to Supplementary Figure 20, 21.

Carbon No.	^{13}C δ (ppm)	HSQC ^{1}H δ (ppm)	*J* value
C1	102.15	4.903 (H1)	d, *J* = 3.4 Hz
C2	73.04	3.326 (H2)	dd, *J* = 10.1 Hz, 3.6 Hz
C3	73.38	3.611-3.587 (H3)	m
C4	81.38	3.391-3.360 (H4)	m
C5	72.64	3.548 (H5)	d, *J* = 9.8 Hz
C6	60.43	3.640 (H6)	s
C1"	152.97	/	/
C2"	109.52	7.429 (H2")	d, *J* = 2.6 Hz
C3"	165.96	/	/
C4"	114.66	7.202 (H4")	dd, *J* = 5.6 Hz, 2.6 Hz
C5"	151.01	8.545 (H5")	d, *J* = 5.6 Hz
C7"	164.22	/	/
C9"	26.51	2.807 (H9")	d, *J* = 4.9 Hz
C11"	148.68-148.57	/	/
C12"	109.74-109.37	7.356 (H12")	dd, *J* = 11.6 Hz, 2.6 Hz
C13"	154.50	/	/
C14"	125.41-125.30	/	/
C15"	123.12	8.178 (H15")	d, *J* = 9.1 Hz
C16"	117.64-117.61	7.090 (H16")	dd, *J* = 8.9 Hz, 1.5 Hz
C18"	152.66-152.06	/	/
C20"	139.47	/	/
C21"	117.15-117.09	8.144 (H21")	s
C22"	127.42-127.12	/	/
C23"	126.63	/	/
C24"	123.49	7.635 (H24")	s
C25"	132.61	7.642 (H25")	s
C26"	121.90	/	/
Oxygen No.	δ (ppm)	^{1}H δ (ppm)	*J* Vaule
O8	/	5.785 (H8)	d, *J* = 7.1 Hz
O9	/	5.805 (H9)	d, *J* = 2.4 Hz,
O11	/	4.573 (H10)	t, *J* = 5.6 Hz
Nitrogen No.	δ (ppm)	^{1}H δ (ppm)	*J* Vaule
N8"	/	8.814 (H8")	q, *J* = 4.6 Hz
N17"	/	8.762 (H17")	s
N19"	/	9.536 (H19")	s

Supplementary Table 7. ^{13}C NMR and ^{1}H NMR chemical shifts of Rho in DMSO-D6. Supplementary Table 7 refers to Supplementary Figure 22, 23.

Carbon No.	^{13}C δ (ppm)	HSQC ^{1}H δ (ppm)	*J* value
C1	131.45	7.019 (H1)	d, *J* = 9.5 Hz
C2	114.92	7.097 (H2)	dd, *J* = 9.5 Hz, 1.7 Hz
C3	155.49	/	/
C4	96.32	6.976 (H4)	d, *J* = 1.8 Hz
C5	96.32	6.976 (H5)	d, *J* = 1.8 Hz
C6	155.49	/	
C7	114.92	7.097 (H7)	dd, *J* = 9.5 Hz, 1.7 Hz
C8	131.45	7.019 (H8)	d, *J* = 9.5 Hz
C9	131.31	/	/
C10	113.32	/	/
C11	157.54	/	/
C13	157.54	/	/
C14	113.32	/	/
C16	45.77	3.647 (H16)	dd, *J* = 13.6 Hz, 6.6 Hz
C17	12.90	1.210 (H17)	t, *J* = 6.8 Hz,
C18	45.77	3.647 (H18)	dd, *J* = 13.6 Hz, 6.6 Hz
C19	12.90	1.210 (H19)	t, *J* = 6.8 Hz,
C21	45.67	3.647 (H21)	dd, *J* = 13.6 Hz, 6.6 Hz
C22	12.90	1.210 (H22)	t, *J* = 6.8 Hz,
C23	45.67	3.647 (H23)	dd, *J* = 13.6 Hz, 6.6 Hz
C24	12.90	1.210 (H24)	t, *J* = 6.8 Hz,
C25	133.68	/	/
C26	130.72	7.474 (H26)	d, *J* = 7.3 Hz
C27	133.13	7.875 (H27)	t, *J* = 7.2 Hz
C28	130.81	7.814 (H28)	t, *J* = 7.4 Hz
C29	131.42	8.232 (H29)	d, *J* = 7.6 Hz
C30	130.66	/	/
C31	166.73	/	/

Supplementary Table 8. ^{13}C NMR and ^{1}H NMR chemical shifts of Rho@γ-CD in DMSO-D6. Supplementary Table 8 refers to Supplementary Figure 26, 27.

Carbon No.	^{13}C δ (ppm)	HSQC ^{1}H δ (ppm)	*J* value
C1	102.18	4.893 (H1)	d, *J* = 3.1 Hz
C2	73.10	3.336-3.310 (H2)	m
C3	73.42	3.597 (H3)	d, *J* = 9.3 Hz
C4	81.42	3.375-3.336 (H4)	m
C5	72.68	3.541 (H5)	d, *J* = 9.8 Hz
C6	60.48	3.622 (H6)	d, *J* = 15.0 Hz
C1"	131.50	7.021 (H1")	d, *J* = 9.5 Hz
C2"	114.94	7.090 (H2")	dd, *J* = 9.5 Hz, 1.6 Hz
C3"	155.52	/	/
C4"	96.36	6.972 (H4")	d, *J* = 1.8 Hz
C5"	96.36	6.972 (H5")	d, *J* = 1.8 Hz
C6"	155.52	/	/
C7"	114.94	7.090 (H7")	dd, *J* = 9.5 Hz, 1.6 Hz
C8"	131.50	7.021 (H8")	d, *J* = 9.5 Hz
C9"	131.35	/	/
C10"	113.35	/	/
C11"	157.58	/	/
C13"	157.58	/	/
C14"	113.35	/	/
C16"	45.81	3.637 (H16")	s
C17"	12.93	1.212 (H17")	t, *J* = 6.9 Hz
C18"	45.81	3.637 (H18")	s
C19"	12.93	1.212 (H19")	t, *J* = 6.9 Hz
C21"	45.81	3.637 (H21")	s
C22"	12.93	1.212 (H22")	t, *J* = 6.9 Hz
C23"	45.81	3.637 (H23")	s
C24"	12.93	1.212 (H24")	t, *J* = 6.9 Hz
C25"	133.76	/	
C26"	130.79	7.473 (H26")	d, *J* = 7.3 Hz
C27"	133.20	7.877 (H27")	t, *J* = 7.3 Hz
C28"	130.86	7.815 (H28")	t, *J* = 7.3 Hz,
C29"	131.41	8.234 (H29")	d, *J* = 7.6 Hz,
C30"	130.70	/	/
C31"	166.82	/	/

Supplementary Table 9. ^{13}C NMR and ^{1}H NMR chemical shifts of Rho@M-γ-CD in DMSO-D6. Supplementary Table 9 refers to Supplementary Figure 29, 30.

Carbon No.	^{13}C δ (ppm)	HSQC ^{1}H δ (ppm)	*J* value
C1	102.18	4.892 (H1)	d, *J* = 3.3 Hz
C2	73.11	3.337 (H2)	s
C3	73.43	3.596 (H3)	d, *J* = 9.1 Hz
C4	81.42	3.375-3.337 (H4)	m
C5	72.68	3.540 (H5)	d, *J* = 9.6 Hz
C6	60.48	3.633 (H6)	s
C10	155.06	/	/
C1'	94.49	4.889 (H1')	s
C2'	71.93	3.346 (H2')	d, *J* = 9.7 Hz
C3'	71.11	3.587 (H3')	s
C4'	67.90	3.365 (H4')	d, *J* = 9.3 Hz
C5'	73.70	3.550 (H5')	s
C6'	62.05	3.619 (H6')	d, *J* = 13.9 Hz
C1"	131.24	6.954 (H1")	d, *J* = 8.2 Hz
C2"	114.89	7.011 (H2")	d, *J* = 6.2 Hz
C3"	154.67	/	/
C4"	96.50	6.911 (H4")	s
C5"	96.50	6.911 (H5")	s
C6"	154.67	/	/
C7"	114.89	7.011 (H7")	d, *J* = 6.2 Hz
C8"	131.15	6.954 (H8")	d, *J* = 8.2 Hz
C9"	131.11	/	/
C10"	113.88	/	/
C11"	157.00	/	/
C13"	157.00	/	/
C14"	113.88	/	/
C16"	45.64	3.633-3.587 (H16")	m
C17"	12.94	1.198 (H17")	t, *J* = 6.9 Hz
C18"	45.64	3.633-3.587 (H18")	m
C19"	12.94	1.198 (H19")	t, *J* = 6.9 Hz
C21"	45.64	3.633-3.587 (H21")	m
C22"	12.94	1.198 (H22")	t, *J* = 6.9 Hz
C23"	45.64	3.633-3.587 (H23")	m
C24"	12.94	1.198 (H24")	t, *J* = 6.9 Hz
C25"	133.50	/	/
C26"	130.81	7.448 (H26")	d, *J* = 7.4 Hz,
C27"	133.50	7.864 (H27")	t, *J* = 7.4 Hz
C28"	130.92	7.801 (H28")	t, *J* = 7.3 Hz
C29"	131.31	8.202 (H29")	d, *J* = 7.7 Hz
C30"	130.72	/	/
C31"	167.12	/	/
Oxygen No.	δ (ppm)	^{1}H δ (ppm)	*J* Vaule
O8	/	5.755 (H8)	s

O9	/	5.778 (H9)	s
O11	/	4.534 (H10)	s

Supplementary Table 10. Half maximal inhibitory concentration (IC50) values for RG, RG@γ-CD and RG@M-γ-CD in CRC cells. Supplementary Table 10 refers to Supplementary Figure 32.

**IC50, μM**	CT26		HT29		SW480		RKO	
**RG**	8 h	12 h	8 h	12 h	8 h	12 h	8 h	12 h
	9.52	8.20	7.65	6.84	8.44	8.08	8.42	7.28
**RG@γ-CD**	8 h	12 h	8 h	12 h	8 h	12 h	8 h	12 h
	6.65	5.76	5.66	4.58	7.20	5.74	7.35	5.05
**RG@M-γ-CD**	8 h	12 h	8 h	12 h	8 h	12 h	8 h	12 h
	5.86	3.04	4.43	2.63	5.15	4.20	5.82	4.45

Supplementary Table 11. IC50 values for RG, RG@γ-CD and RG@M-γ-CD in target cells. Supplementary Table 11 refers to Supplementary Figure 33.

**IC50, μM**	HUVECs		PMs		DCs		splenocytes	
**RG**	12 h	24 h	12 h	24 h	12 h	24 h	12 h	24 h
	4.66	2.54	9.22	4.68	/	9.24	/	/
**RG@γ-CD**	12 h	24 h	12 h	24 h	12 h	24 h	12 h	24 h
	3.75	2.62	5.08	4.20	/	8.14	/	9.65
**RG@M-γ-CD**	12 h	24 h	12 h	24 h	12 h	24 h	12 h	24 h
	3.48	1.76	4.34	4.06	/	7.60	9.34	8.50

Supplementary Table 12. Sequence of primers used for quantitative real-time PCR. PMs seeded in 6-well plates (1×10^6 cells/well) were treated with different formulations for 24 h, then incubated with LPS (10 ng/mL) or IL-4 (40 ng/mL) for 2 h. Quantitative real-time PCR was performed with the treated PMs using primers below to determine the mRNA expression level of phenotype-related cytokines.

mRNA	Primers (5'-3')
IL-1β	F: TGTGAAATTGCCACCTTTTGA
	R: TGTCCTCATCCTGGAAGGTC
IL-6	F: CTGCAAGAGACTTCCATCCAG
	R: AGTGGTATAGACAGGTCTGTTGG
TNF-α	F: GAACTGGCAGAAGAGGCACT
	R: AGGGTCTGGGCCATAGAACT
TGF-β1	F: GGAGAGCCCTGGATACCAAC
	R: CAACCCAGGTCCTTCCTAAA
Arg-1	F: AGAGCTGACAGCAACCCTGT
	R: GGATCCAGAAGGTGATGGAA
IL-10	F: GCTGGACAACATACTGCTAACC
	R: ATTTCCGATAAGGCTTGGCAA
CD206	F: CTCTGTTCAGCTATTGGACGC
	R: TGGCACTCCCAAACATAATTTGA
VEGF-B	F: GCCAGACAGGGTTGCCATAC
	R: GGAGTGGGATGGATGATGTCAG
PDGF-α	F: TGGCTCGAAGTCAGATCCACA
	R: TTCTCGGGCACATGGTTAATG
MMP-9	F: GGACCCGAAGCGGACATTG
	R: CGTCGTCGAAATGGGCATCT
GAPDH	F: ACCCAGAAGACTGTGGATGG
	R: CTTGCTCAGTGTCCTTGCTG

Supplementary Table 13. Antibodies for immunohistochemistry and FACS.

Western blot		
anti-pVEGFR-2	CST, 3817	1:3000
anti-VEGFR-2	CST, 9698	1:2000
anti-pPDGFR-β	CST, 2227	1:2000
anti-PDGFR-β	CST, 3169	1:2000
anti-pP65	Santa Cruz, sc-136548	1:1000
anti-P65	Abcam, ab16502	1:1000
anti-ABC1	Santa Cruz, sc-58219	1:1000
anti-pERK	CST, 4370S	1:2000
anti-ERK	CST, 4695S	1:1000
anti-GAPDH	CST, 5174	1:3000
Immunohistochemistry		
TUNEL	Roche, 39081800	not diluted
anti-Ki67	Abcam, ab16667	1:200
anti-mouse CD34	Abcam, ab81289	1:50
anti-mouse VEGF	Abcam, ab46154	1:50
Immunofluorescence		
DAPI	Abcam, ab104139	not diluted
Anti-mouse F4/80	CST, 70076	1:100
Anti-mouse CD206	Santa Cruz, sc-58986	1:50
Anti-mouse CD80	Santa Cruz, sc-376012	1:100
FACS		
APC anti-mouse F4/80	Biolegend, 123116	1:200
PerPC anti-mouse/human CD11b	Biolegend, 101229	1:200
FITC anti-mouse CD206	Biolegend, 141703	1:200
PE anti-mouse CD80	Biolegend, 104707	1:200
APC/Cyanine7 anti-mouse CD45	Biolegend, 103116	1:200
